# Supplementary material for: Continued attendance in a PrEP program despite low adherence and non-protective drug levels among adolescent girls and young women in Kenya: Results from a prospective cohort study
Source: PLoS Med. 2022 Sep 12;19(9):e1004097. doi: 10.1371/journal.pmed.1004097 (PMC9521917; doi:10.1371/journal.pmed.1004097)
Supplement: S1 STROBE Checklist — (PDF) [file pmed.1004097.s001.pdf]

S1 STROBE. STROBE check list

	Item No.	Recommendation	Page No. & section	Relevant text from manuscript
**Title and abstract**	1	(*a*) Indicate the study's design with a commonly used term in the title or the abstract	1	**prospective cohort study**
		(*b*) Provide in the abstract an informative and balanced summary of what was done and what was found	3-4<br>Abstract	336 adolescent girls and young women (AGYW) participating in the PEPFAR-funded DREAMS Initiative in western Kenya were enrolled into a study of PrEP use conducted from 6/2019 to 1/2020. AGYW in the DREAMS PrEP program, which used daily oral TDF/FTC completed interviews, and provided dried blood spots for measurement of tenofovir-diphosphate (TFV-DP) concentrations, a measure of adherence over weeks-months, at enrolment and three months later. Among AGYW who reported they were continuing PrEP, >90% indicated they were using PrEP to prevent HIV, although almost all had TFV-DP levels that were non-protective. Many AGYW persisted in the PrEP program without taking PrEP frequently enough to receive benefit.
**Introduction**				
Background/rationale	2	Explain the scientific background and rationale for the investigation being reported	6<br>Introduction Paragraph 3	Importantly, many women in clinical trials of daily oral PrEP in SSA continued to attend study visits but did not adhere to daily dosing.
Objectives	3	State specific objectives, including any prespecified hypotheses	7, 8<br>Introduction Paragraph 6<br><br>Methods Paragraph 3	The main questions examined were the extent of program persistence (a measure focusing on visit attendance), and the level of protection from HIV infection among program attendees as assessed by measuring PrEP metabolites in blood.<br><br>This analysis focuses on two study objectives: measurement of PrEP adherence by self-report and biomarkers, and

				identification of factors associated with PrEP persistence and adherence.
**Methods**				
Study design	4	Present key elements of study design early in the paper	7 Methods paragraph 1	A prospective study was conducted from 6/2019 - 1/2020 among AGYW participants in the DREAMS PrEP program. This study included face-to-face interviews at enrolment and 3 months later, and DBS sample collection for assays of intracellular drug metabolites (tenofovir diphosphate, TVF-DP, as an objective measure of PrEP adherence) from AGYW who reported PrEP use at the time of interview.
Setting	5	Describe the setting, locations, and relevant dates, including periods of recruitment, exposure, follow-up, and data collection	7, methods paragraph 1	conducted from 6/2019 - 1/2020<br>residing in Kisumu and Homa Bay counties
Participants	6	(*a*) *Cohort study*—Give the eligibility criteria, and the sources and methods of selection of participants. Describe methods of follow-up<br>*Case-control study*—Give the eligibility criteria, and the sources and methods of case ascertainment and control selection. Give the rationale for the choice of cases and controls<br>*Cross-sectional study*—Give the eligibility criteria, and the sources and methods of selection of participants	7-8 Methods paragraph 2	From the DREAMS database, we identified 613 AGYW who met our eligibility criteria of 18-to-24 years old, residing in Kisumu and Homa Bay counties, enrolled in the PrEP program for 2-9 months with a visit for PrEP initiation or refill between October 2018 and April 2019, and had returned for a refill in the two months prior to study start in June 2019 (to exclude AGYW who might have recently stopped using PrEP). From the 613 eligible AGYW, we randomly sampled (using the 'sample' command in STATA) to select and enroll 359 AGYW who met the above eligibility criteria, were able to speak English, Dholuo, or Kiswahili and had indicated on the DREAMS enrollment consent form willingness to be contacted for future studies.
		(*b*) *Cohort study*—For matched studies, give matching criteria and number of exposed and unexposed<br>*Case-control study*—For matched studies, give matching criteria and the number of controls per case		
Variables	7	Clearly define all outcomes, exposures, predictors, potential confounders, and effect modifiers. Give diagnostic criteria, if applicable	8-9 Methods paragraph 8	The main outcome of interest in this study is PrEP persistence. Persistent AGYW were defined as having attended both interviews as well as interim visits for PrEP refills and stating

			Methods paragraph 5	that they were taking PrEP at both interviews. A secondary outcome is PrEP adherence based on the TFV-DP levels from the testing of the DBS samples. Adherent AGYW were defined as having TFV-DP levels of 700+ fmol/punch, the equivalent of 4+ doses per week taken regularly[31]. We included cut-offs at 350 fmol/punch, (~one to two days per week) as well as the lower limit of quantification (200 fmol/punch) and the lower limit of detection (10 fmol/punch); levels below 10 were taken as consistent with no PrEP use in the recent past.<br><br>We conducted one-on-one in-person structured interviews with all participants in their preferred language, entering data electronically via Open Data Kit (ODK). The instrument captured information on: 1) socio-demographic characteristics (including age, marital status, current school attendance, living condition), 2) participation in and perceptions of the DREAMS program (including being in a PrEP support group, being active in the DREAMS program), 3) experience with PrEP and support for PrEP use among partners, family members and the community, 4) self-reported continued use of and adherence to PrEP, 5) HIV risk perception, 6) contraceptive use, 7) alcohol use (AUDIT17), 8) intimate partner violence (HITS, 4 items19), 9) social support (MOS Social Support Scale, 19 items) 20, 10) depression (PHQ-9, 10 items 21) and used scales adapted for previously used in Kenya or other African countries (ref 17, 19, 20, 21).
			Methods paragraph 7	In addition to the study data collected at the two interviews, pharmacy PrEP refill information was obtained from the DREAMS program data.
Data sources/ measurement	8*	For each variable of interest, give sources of data and details of methods of assessment (measurement). Describe comparability of assessment methods if there is more than one group	*8,9* Methods paragraph 5	The instrument captured information on: 1) socio-demographic characteristics (including age, marital status, current school attendance, living condition), 2) participation in and perceptions of the DREAMS program (including being active in the DREAMS program), 3) experience with PrEP and support for PrEP use among partners, family members and the community (e.g. being in a PrEP support group, have told partner, family members and friends of PrEP use, partner support of PrEP use, have friends on PrEP) 4) self-reported continued use of and

				adherence to PrEP (e.g. number of PrEP pills taken in the past week or month), 5) HIV risk perception (e.g. feeling that partner behavior put AGYW at risk, perception of being at moderate to high HIV risk if not taking PrEP), 6) condoms use and contraceptive use, 7) alcohol use (AUDIT17), 8) intimate partner violence (HITS, 4 items19), 9) social support (MOS Social Support Scale, 19 items) 20, 10) depression (PHQ-9, 10 items 21) and used scales previously used in Kenya or other African countries (ref 17, 19, 20, 21).
			Methods paragraph 7	TFV-DP was analyzed using a validated liquid chromatography mass spectrometry (LC-MS/MS) with calibration curve range of 200-10,000 fmol/3mm punch. Internal/external quality control samples were included in each run, with external samples cross-validated with intra-laboratory testing. In addition to the study data collected at the two interviews, pharmacy PrEP refill information was obtained from the DREAMS program data.
			Methods paragraph 8	The main outcome of interest in this study is PrEP persistence. Persistent AGYW were defined as having attended both interviews as well as interim visits for PrEP refills and stating that they were taking PrEP at both interviews. A secondary outcome is PrEP adherence based on the TFV-DP levels from the testing of the DBS samples. Adherent AGYW were defined as having TFV-DP levels of 700+ fmol/punch, the equivalent of 4+ doses per week taken regularly22
Bias	9	Describe any efforts to address potential sources of bias	9 Methods Paragraph 9	We used univariable and multivariable (adjusted) generalized estimating equations modification of logistic regression models accounting for clustering of AGYW within wards to assess the associations between persistence and factors measured at Interview 2.
Study size	10	Explain how the study size was arrived at	8 Methods Paragraph 3	This sample size assumed a discontinuation or loss to follow-up rate of 50%, and a two-level factor potentially associated with PrEP adherence. Our sample size was sufficient to detect a minimum absolute difference of 15% in the PrEP adherence

				rates at the first interview between the levels of the factor at 80% power given a type I error rate of 5% and a two-sided test.
Quantitative variables	11	Explain how quantitative variables were handled in the analyses. If applicable, describe which groupings were chosen and why	9 Methods Paragraph 9	We summarized categorical variables as frequency and percentages and continuous variables as mean and standard deviation (SD) or median and interquartile range (IQR). We used univariable and multivariable (adjusted) generalized estimating equations modification of logistic regression models accounting for clustering of AGYW within wards to assess the associations between persistence and characteristics factors measured at Interview 2.
Statistical methods	12	(*a*) Describe all statistical methods, including those used to control for confounding	9 Methods Paragraph 9	We used univariable and multivariable (adjusted) generalized estimating equations modification of logistic regression models accounting for clustering of AGYW within wards to assess the associations between persistence and factors measured at Interview 2. The multivariable models adjusted for county of residence and factors that had a p-value<0.1 in the univariable analysis.
		(*b*) Describe any methods used to examine subgroups and interactions		
		(*c*) Explain how missing data were addressed	9 Methods Paragraph 9	The proportion of AGYW who attended Interview 2 and had missing information on factors of interest was <10%. The analyses were based on complete case data assuming missing completely at random for the missing data mechanism.
		(*d*) *Cohort study*—If applicable, explain how loss to follow-up was addressed<br>*Case-control study*—If applicable, explain how matching of cases and controls was addressed<br>*Cross-sectional study*—If applicable, describe analytical methods taking account of sampling strategy	9 Methods Paragraph 9	AGYW who were lost to follow up or censored due to study closure prior to Interview 2 were excluded from the analysis
		(*e*) Describe any sensitivity analyses		

**Results**				
Participants	13*	(a) Report numbers of individuals at each stage of study—e.g., numbers potentially eligible, examined for eligibility, confirmed eligible, included in the study, completing follow-up, and analysed	(10)	Figure 1
		(b) Give reasons for non-participation at each stage	10 Results Paragraph 2	Of the 336 AGYW taking PrEP at enrollment, 302 attended Interview2, with 18 lost to follow-up and 16 censored. At Interview2, 105 said they had discontinued PrEP and 197 said they were continuing PrEP (Figure 1). Most (246/302, 81%) AGYW interviewed at follow-up had attended at least a PrEP dispensation visit between the two interviews. Fifty-six had no record of attendance at interim PrEP refill visits, including 21 of the 197 AGYW who reported they were continuing PrEP. This left 176 of the AGYW who attended Interview2 (176/302 58.3%, 95%CI [52,7%, 63.8%]) who met our definition of PrEP persistence.
		(c) Consider use of a flow diagram	(10)	Figure 1
Descriptive data	14*	(a) Give characteristics of study participants (e.g., demographic, clinical, social) and information on exposures and potential confounders	(10)	Table 1
		(b) Indicate number of participants with missing data for each variable of interest		18 participants were lost to follow up and did not attend Interview2. Hence, they had missing information on all variables measured at Intervew2. Among the 302 participants who attended Interview2, the

				proportion who had missing information on the variables of interest was<10%.
		(c) *Cohort study*—Summarise follow-up time (e.g., average, and total amount)	10 Results Paragraph 2	The median time between the two interviews was 93 (IQR [91,99]) days.
Outcome data	15*	*Cohort study*—Report numbers of outcome events or summary measures over time	10 Results Paragraph 2	Of the 336 AGYW taking PrEP at enrollment, 302 attended Interview2, with 18 lost to follow-up and 16 censored. At Interview2, 105 said they had discontinued PrEP and 197 said they were continuing PrEP (Figure 1). Most (246/302, 81%) AGYW interviewed at follow-up had attended at least a PrEP dispensation visit between the two interviews. Fifty-six had no record of attendance at interim PrEP refill visits, including 21 of the 197 AGYW who reported they were continuing PrEP. This left 176 of the AGYW who attended Interview2 (176/302) 58.3%, 95%CI [52,7%, 63.8%]) who met our definition of PrEP persistence.
		Case-control study—Report numbers in each exposure category, or summary measures of exposure		
		Cross-sectional study—Report numbers of outcome events or summary measures		
Main results	16	(*a*) Give unadjusted estimates and, if applicable, confounder-adjusted estimates and their precision (e.g., 95% confidence interval). Make clear which confounders were adjusted for and why they were included	12-13	Tables 2 and 3

		(*b*) Report category boundaries when continuous variables were categorized	9 Methods Paragraph 8	Adherent AGYW were defined as having TFV-DP levels of 700+ fmol/punch, the equivalent of 4+ doses per week taken regularly ([31]). We included cut-offs at 350 fmol/punch, (~one to two days per week) [31], as well as the lower limit of quantification (200 fmol/punch) and the lower limit of detection (10 fmol/punch); levels below 10 were taken as consistent with no PrEP use in the recent past
		(*c*) If relevant, consider translating estimates of relative risk into absolute risk for a meaningful time		
Other analyses	17	Report other analyses done—e.g., analyses of subgroups and interactions, and sensitivity analyses		
**Discussion**				
Key results	18	Summarise key results with reference to study objectives	12 Discussion Paragraph 1	In this study designed to examine PrEP adherence, persistence and factors associated with persistence, nested within a real-world PrEP program in western Kenya, AGYW had moderate retention in the program, and most reported high adherence and continuation of oral PrEP. However, a minority had detectable TFV-DP at both interview visits, and only a small percentage achieved or sustained sufficient drug concentrations to prevent HIV acquisition. Most (85%) continued to receive HIV prevention support through the DREAMS initiative. Notably, AGYW who persisted in the PrEP program had a higher self-perceived risk of HIV infection. Among AGYW who reported they were continuing

				PrEP at the second interview, over 90% indicated the reason was to prevent HIV, although almost all had non-protective TFV-DP levels.
Limitations	19	Discuss limitations of the study, taking into account sources of potential bias or imprecision. Discuss both direction and magnitude of any potential bias	14 Discussion Paragraph 5	Limitations of this study include both size and generalizability, as we included a relatively small sample size from a large programmatic project, which may be most generalizable to AGYW in western Kenya. In addition, all AGYW in the study were recruited from the DREAMS initiative and were receiving support for HIV prevention that would likely not be available to them otherwise. Other limitations include potential social desirability bias, recall error in the self-reported data and possibly remaining confounding bias.
Interpretation	20	Give a cautious overall interpretation of results considering objectives, limitations, multiplicity of analyses, results from similar studies, and other relevant evidence	15 Discussion Paragraph 6	In conclusion, our study provides insight into oral PrEP use in a real-world programmatic setting, as well as predictors of PrEP persistence in this context. The results reveal that most AGYW may be better protected by long-acting injectable PrEP than by oral daily PrEP. Future research is needed to clarify whether persistence without adequate adherence is as common among AGYW in other settings, and whether new long-acting PrEP formulations, adequately supported by facilitators identified during use of existing PrEP agents, can afford higher level protection to this very vulnerable population.

Generalisability	21	Discuss the generalisability (external validity) of the study results	14<br>Discussion<br>Paragraph 5	Limitations of this study include both size and generalizability, as we included a relatively small sample size from a large programmatic project, which may be most generalizable to AGYW in western Kenya.
**Other information**				
Funding	22	Give the source of funding and the role of the funders for the present study and, if applicable, for the original study on which the present article is based	15	The study is supported by NIH grants R01HD094682 and 3R01HD094682-02S1. Funders were not involved in study design, execution, or interpretation of results.

*Give information separately for cases and controls in case-control studies and, if applicable, for exposed and unexposed groups in cohort and cross-sectional studies.

Note: An Explanation and Elaboration article discusses each checklist item and gives methodological background and published examples of transparent reporting. The STROBE checklist is best used in conjunction with this article (freely available on the Web sites of PLoS Medicine at http://www.plosmedicine.org/, Annals of Internal Medicine at http://www.annals.org/, and Epidemiology at http://www.epidem.com/). Information on the STROBE Initiative is available at www.strobe-statement.org
