# Supplementary material for: Continued attendance in a PrEP program despite low adherence and non-protective drug levels among adolescent girls and young women in Kenya: Results from a prospective cohort study
Source: PLoS Med. 2022 Sep 12;19(9):e1004097. doi: 10.1371/journal.pmed.1004097 (PMC9521917; doi:10.1371/journal.pmed.1004097)
Supplement: S1 Protocol — (DOCX) [file pmed.1004097.s002.docx]

###

### S1 Protocol. The protocol of the ”PrEP Adherence for Kenyan adolescent girls and young women” study

**Title:** **PrEP Adherence for Kenyan adolescent girls and young women**

**Sponsor:** Fred Hutchinson Cancer Research Center

**Principal Investigator:** Jane Cover, PhD MPH

**Site** **Principal Investigator**: Christopher Obong’o, PhD

**Co-Investigators:** Paul Drain, MD PhD

Martha Brady, MS

Rael Obanda, MHIM

Edward Kariithi, MBcHB

James Mukabi, MBcHB

Juma Mwatsefu, MA

Oluoch Madiang, Bed

**Version** 9

**Table of Contents**

[List of Abbreviations 3](#_Toc22897751)

[1.0 Background and rationale for the study 4](#_Toc22897752)

[2.0 Study objectives 9](#_Toc22897753)

[3.0 Study population, study design, and sample size 10](#_Toc22897754)

[4.0 Study procedures 15](#_Toc22897755)

[5.0 Eligibility criteria 17](#_Toc22897756)

[6.0 Recruitment 18](#_Toc22897757)

[7.0 Illustrative lines of inquiry 19](#_Toc22897758)

[8.0 Consent process 21](#_Toc22897759)

[9.0 Risks 22](#_Toc22897760)

[10.0 Mitigation of risks 23](#_Toc22897761)

[11.0 Benefits 23](#_Toc22897762)

[12.0 Study and safety monitoring 24](#_Toc22897763)

[13.0 Managing and reporting adverse events 24](#_Toc22897764)

[14.0 Managing and reporting unanticipated problems or protocol deviations 24](#_Toc22897765)

[15.0 Confidentiality and data management 24](#_Toc22897766)

[16.0 Study costs 26](#_Toc22897767)

[17.0 Care for injury 26](#_Toc22897768)

[18.0 Compensation 26](#_Toc22897769)

[19.0 Investigator responsibilities 26](#_Toc22897770)

### List of Abbreviations

AGYW – Adolescent girls and young women

ART – Anti retroviral therapy

DBS – Dried blood spot

DREAMS Initiative - Determined, Resilient, Empowered, AIDS-free, Mentored, and Safe Initiative

FGD – Focus group discussion

FTC - Emtricitabine

HIV – Human Immunodeficiency Virus

HTS – HIV Testing Services

IDI – In depth interview

MSM – Men who have sex with men

PEPFAR - President’s Emergency Plan for AIDS Relief

PrEP - HIV pre-exposure prophylaxis

REC – Research Ethics Committee

RDC – Research Determination Committee

SSA – sub-Saharan Africa

TFV – Tenofovir

TFV-DP – Tenofovir Diphosphate

### 1.0 Background and rationale for the study

**HIV and Adolescent Girls and Young Women**

In 2014, there were approximately 880 million adolescent girls and young women (AGYW) aged 15-24 years in the world, comprising 12% of the total population.^[[1]](#endnote-1)^ Overall, 15% of women living with HIV worldwide are aged 15-24 years, with the majority (80%) living in sub-Saharan Africa (SSA).1 These young women experience among the highest HIV incidence rates in the world. An estimated 380,000 adolescent girls and young women are infected with HIV every year.

Adolescent girls and young women have up to 8 times the risk of HIV infection compared to their male counterparts.^[[2]](#endnote-2)^ A consistent pattern has been seen in many countries in SSA for over a decade - while HIV infection in adolescent boys and young men is fairly low, HIV incidence rises rapidly in young girls at the age of sexual debut and increases steadily until ages 20-30 (see data from South Africa in Table 1).^[[3]](#endnote-3)^ Many SSA countries show a similar pattern; for example, in Uganda, Swaziland, Mozambique and Kenya the HIV prevalence in AGYW 15-24 years old is 1.8, 1.7, 2.3 and 1.6-fold higher for females relative to their male counterparts. While the magnitude of these early differences decreases over time as HIV prevalence in men increases with age, the overall prevalence of HIV infection in women remains higher than that in men in most countries.

Table 1: HIV prevalence amongst high school students in rural Kwazulu-Natal

| Age group | Percent HIV Prevalence (2010), with 95% Confidence Interval | | |
| --- | --- | --- | --- |
|  | Male (n=1252) | Female (n=1423) | Female : Male ratio |
| < 15 years | 1.0 | 2.6 | 2.6 |
| 16-17 years | 1.1 | 6.1 | 5.5 |
| 18-19 years | 1.5 | 13.6 | 9.1 |
| 20 + years | 1.8 | 24.7 | 13.7 |

**The DREAMS Initiative in Kenya**

Several programs have been launched to address the ongoing high HIV risk among AGYW in SSA. The most ambitious among them is the DREAMS (Determined, Resilient, Empowered, AIDS-free, Mentored, and Safe) Initiative, which is a partnership of private industry with PEPFAR that seeks to reduce HIV infections among AGYW in 10 sub-Saharan African countries that account for almost 50% of the new infections in girls and women worldwide.^[[4]](#endnote-4)^ The DREAMS prevention package is built on the hypothesis that the impact of multiple simultaneous interventions will be synergistic and will result in the greatest reduction in outcomes such as HIV incidence, unintended pregnancy and school drop-out. This layered intervention package focuses on 4 areas: empowering AGYW and reducing their risk, reducing risk in their sexual partners, strengthening families, and mobilizing communities for change. It includes increased access to contraceptives and condoms, community support and interventions against gender-based violence, anti-retroviral therapy (ART) and voluntary medical circumcision for young men, cash transfers to promote continuation in school and access to Pre-Exposure Prophylaxis or PrEP.

In Kenya, while remarkable progress has been made in reducing overall HIV incidence, young women ages 15-24 years continue to bear a disproportionate burden of new infections.1^,^2 In 2015, young women ages 18-24 years accounted for >30% of all new infections.2 PATH leads the implementation of the PEPFAR-funded DREAMS Initiative in Kisumu and Homabay counties in the Western Kenya region. In sum, about 10500 new HIV infections were reported in young people ages 15-24 years in these two counties in 2015, accounting for 14% national HIV incidence.2 The DREAMS Initiative, a multi-component HIV prevention program, focuses on empowering AGYW and reducing their risk, reducing risk in their sexual partners, strengthening families, and mobilizing communities for change. Efforts have been underway to collect programmatic data on the multi-component intervention programs, which provide real-world opportunities for analysis of the multi-level factors influencing uptake and adherence to programs targeting AGYW. To date a total of 50,299 AGYW in the two counties have been enrolled in the DREAMS program. Many of the interventions for the AGYW are delivered through ‘safe spaces’ which are generally private houses or community centers located in communities throughout the project area where girls can meet with their peers under the mentorship of an adolescent leader.

Beginning in March 2017, AGYW ages 18 to 24 years who are enrolled in DREAMS and meet eligibility requirements, are being offered the opportunity to begin HIV PrEP. The approach to introducing and enrolling AGYW in the PrEP program is illustrated in the DREAMS PrEP Initiation Flow Chart (Attachment A). The idea of PrEP for HIV prevention may be introduced to AGYW ages 18+ via providers or through PrEP sensitization sessions that are part of the DREAMS program. Those interested are then screened for PrEP eligibility, consistent with the following criteria:

• Sexual partner is HIV+

• Sexual partner of unknown status and at high risk

• Transactional sex

• Recent STI

• Recurrent use of PEP

• Sex use while taking alcohol /other drugs

• Injection drug use

• Sero-discordant

Those eligible are tested for HIV infection and those testing negative who wish to take PrEP are enrolled in the program. Adherence counseling is a component of the DREAMS program.

As of November 2017, an estimated 1060 AGYW had enrolled in PrEP in the two counties. As part of the DREAMS implementation, an extensive monitoring and evaluation (M&E) system is in place to collect comprehensive data on each AGYW and her participation in the program. Data collected includes uptake and participation in the different economic and socio-behavioral interventions, biomedical interventions such as HIV and STI testing, use of contraception and PrEP. Data forms include a PrEP eligibility checklist, health provider forms that are completed at each provider visit to the safe spaces and PrEP discontinuation forms that are administered to each AGYW who discontinues PrEP. A service utilization form is also part of the M&E program. This form details the HIV prevention programs that each AGYW participates in (e.g. peer support groups, income generation, etc.) and includes all biomedical services she receives in at the safe space or via referral.

Considering that no single biomedical, behavioral or structural intervention is highly effective for AGYW, the most effective approach to HIV prevention will likely involve multiple interventions in combination. The success of combination prevention interventions depends on both the efficacy and the acceptability of the components for individuals and society, as both factors can affect uptake and adherence and determine the feasibility of broad implementation.

This study will collect supplemental data that is not currently collected in the DREAMS program to provide more context and insights into factors affecting participation in DREAMS and the PrEP program, as well as PrEP adherence and persistence. Specifically, this study will solicit through interviews additional information about the background of participating AGYW, their participation in DREAMS, their motivations for taking or dropping out of PrEP, and the challenges they face in adhering to the PrEP regimen. In addition, because the DREAMS data does not contain valid data on PrEP adherence, this study will also collect biomarkers for detection of tenofovir levels.

**PrEP for HIV prevention**

The potential effectiveness of oral antiretroviral medications to prevent the sexual acquisition of HIV (PrEP) has been well established in placebo-controlled, randomized clinical trials and open label studies. While most of these studies have been in men who have sex with men (MSM) and transgender women,^[[5]](#endnote-5)-^^[[6]](#endnote-6)^^[[7]](#endnote-7)^ efficacy has also been demonstrated in heterosexual men and women in two studies.^[[8]](#endnote-8),^^[[9]](#endnote-9)^ As one would expect, efficacy depends on adherence. Although estimates of efficacy are close to 100% if blood drug levels are consistent with ≥4 doses/week,5^,^^[[10]](#endnote-10),^^[[11]](#endnote-11)^ no efficacy was seen in several studies among women where adherence was low.10^,^^[[12]](#endnote-12)^

Multiple randomized clinical trials have shown that daily oral tenofovir-based PrEP reduces the incidence of HIV among high-risk populations.^[[13]](#endnote-13)-^^[[14]](#endnote-14)^^[[15]](#endnote-15)^^[[16]](#endnote-16)^ The variation in efficacy across PrEP trials has been explained by differences in drug adherence.^[[17]](#endnote-17)-^^[[18]](#endnote-18)^^[[19]](#endnote-19)^ In two PrEP studies that did not show HIV protection (FEM-PrEP and VOICE), measurement of plasma tenofovir levels revealed poor drug adherence with concentrations too low to prevent HIV acquisition.18^,^19 Measurement of intracellular tenofovir-diphosphate (TFV-DP) in peripheral blood mononuclear cells (PBMCs) was adopted as a marker of tenofovir-based PrEP adherence, and a TFV-DP concentration of 16 fmol/108 PBMCs was associated with a 90% reduction in risk of HIV acquisition **(Figure 1)**.1111 While PrEP is clearly efficacious for people who maintain adherence, ensuring drug adherence remains a critical challenge.


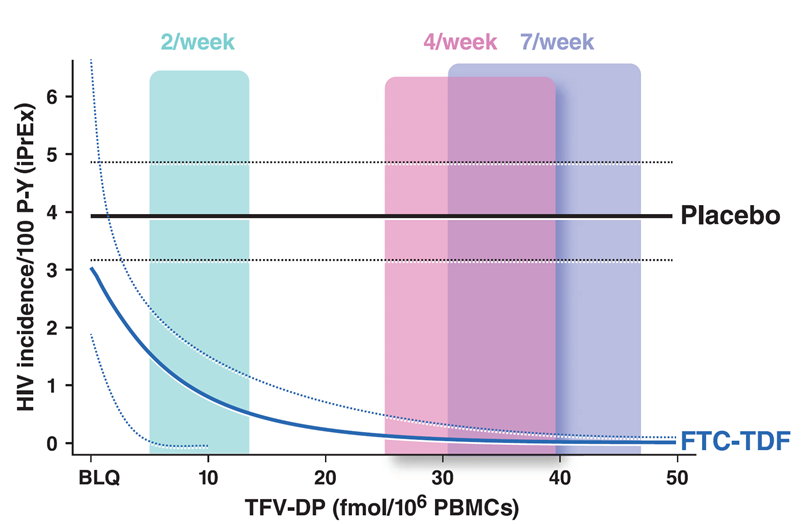


**Figure 1.** Relation of PrEP adherence & HIV acquisition.11

**Figure 1.** Relation of PrEP adherence & HIV acquisition [11].

**Durable PrEP/ART Adherence Measures**

Multiple studies have revealed significant decreases in adherence over longer periods of observation^[[20]](#endnote-20),^^[[21]](#endnote-21)^and among different populations, including high risk women,18^,^19 African American men who have sex with men (MSM), younger MSM21^,^^[[22]](#endnote-22)^and transgender women.^[[23]](#endnote-23)^ The existing tools to monitor ART or PrEP adherence have been inaccurate and too costly for routine clinical use. Patient self-reporting is often ineffective,^[[24]](#endnote-24),^^[[25]](#endnote-25)^ while pill counting and home-based visits for pill monitoring have no association with ART adherence.^[[26]](#endnote-26)-^^[[27]](#endnote-27)^^[[28]](#endnote-28)^ Electronic pill bottle caps/boxes are more accurate, but their high cost and complexity confine their utility to research studies. In resource-limited settings, practitioners often rely on a clinical assessment, which can lead to inappropriate ART regimen switching.24^,^25^,^^[[29]](#endnote-29)^ Monitoring PrEP adherence is even more challenging, since there are no clinical features to assess or monitor.^[[30]](#endnote-30)^ Therefore, new approaches are needed to accurately monitor PrEP and ART adherence. Specifically, an inexpensive, rapid assay to measure HIV drug levels at the clinical point of care has been developed, which may help identify those people who could benefit from additional adherence counseling and targeted support.

**HIV Drug Levels as a Reliable and Acceptable Tool for Monitoring Adherence**

A drug concentration is a more objective measure of adherence, and closely correlates with the clinical outcomes of PrEP^[[31]](#endnote-31),^^[[32]](#endnote-32)^ and ART users.^[[33]](#endnote-33)^ Drug levels in various specimens, including PBMCs,11 plasma/dried blood spots (DBS),^[[34]](#endnote-34)^ and hair,^[[35]](#endnote-35)^ provide more reliable estimates of adherence than patient self-reported adherence.24^,^25 Furthermore, low plasma drug concentrations are associated with virologic failure and HIV transmission.^[[36]](#endnote-36),^[^47-49^](#_ENREF_37) Currently, drug measurement is primarily performed using liquid-chromatography mass-spectrometry (LC-MS/MS) assays, which are expensive and not suitable for routine use in either resource-rich or -poor settings. Due to the reliance on this expensive, complicated technology, the current application of detecting drug levels has been limited to participants of clinical trials. Two recent qualitative studies among PrEP users have used HIV drug levels to explore compliance problems and enhance medication adherence.^[[37]](#endnote-37),^^[[38]](#endnote-38)^ In these retrospective studies, PrEP clients have appreciated receiving information on their drug levels, and many participants expressed a desire for real-time drug concentration monitoring and feedback.^37,^38 In this study, we will be using a simple POC assay developed by PHPT lab/University of Washington aimed at detecting tenofovir concentrations in urine, fingerstick blood, and/or oral fluid.

Results from studies with a particular focus on HIV oral PrEP among adolescents are still pending publication. Preliminary results from conference proceedings^[[39]](#endnote-39)^ indicate that adherence to PrEP decreased over time and suggest that monthly study visits may support greater adherence. Text reminders and participation in adherence clubs with counseling may also improve adherence. Some of these interventions (regular adherence counseling and visits) are explicit components of the DREAMS Program in Kenya. This study will ask participants about the impact of these components.

Auerbach and Hoppe^[[40]](#endnote-40)^ note that a challenge to PrEP adherence in the USA was posed by the perception that taking Truvada indicates that the individual is HIV+, as opposed to HIV- and taking Truvada prophylactically. Consistent with this article, study instruments will include questions exploring ‘risk compensation’ i.e., decreasing condom use with PrEP uptake. Consistent with authors Venter et al.^[[41]](#endnote-41)^, this study will pose a number of questions exploring the advantages and disadvantages of provision of PrEP in community settings (as opposed to busy health facilities), to understand its feasibility and potential impact on adherence. Specifically with respect to adolescents, the same authors propose making PrEP available in ‘adolescent shared spaces’, which is consistent with the DREAMS approach. More generally, this study design and instruments reflect the perspective of these authors that differences in adherence, especially among youth, may reflect “differences in access to health services, provider attitudes about sexuality, self-agency and influence of peers”. Finally, the study approach (and instruments) reflects many of the factors identified by Celum et al.^[[42]](#endnote-42)^ as worthy of future exploration, specifically: whether low adherence reflects low HIV risk perception; lack of self-efficacy; stigma; an inability to take a daily pill; a lack of motivation and interest in HIV prevention; concerns about drug side effects; and negative reactions and lack of peer, family and/or partner support.

Amico et al. (2016)^[[43]](#endnote-43)^ note that there have been limited evaluations of the accuracy of self-reported PrEP adherence in open label trials and implementation studies. They suggest that adherence in these types of studies may differ from placebo-controlled trials. For example, a qualitative study conducted during the FEM-PrEP found over-reporting due to fears of being terminated from the trial (Corneli 2015)^[[44]](#endnote-44)^ while in open label projects and real-world PrEP implementation, termination from a trial or program may be less of a pressure. However social desirability bias remains a challenge. In their open-label trial, Amico et al (2016) found that self-reported recent PrEP use had good correlation with the biomarker measures, yet they also found 17% of participants over-reported adherence, associated with lower age and education.

Tenofovir disoproxil fumarate (TDF) has good pharmacokinetic properties for objectively measuring adherence.^[[45]](#endnote-45)^ Liquid chromatography and tandem mass spectrometry (LC-MS/MS) is an established platform for this type of testing. TFV has a plasma elimination half-life of 17 hours, while TFV-DP has an intracellular half-life of 4.2 days in PBMCs and 17.1 days in red blood cells.34 The different half-lives of these drug forms are advantageous for assessing recent and cumulative adherence within a POC test.

By design, this study focuses on older adolescents and young women, who are the target population for PrEP enrollment via the DREAMS Initiative, and, as described above, are particularly vulnerable to HIV acquisition. It is expected that this study will enable PATH and other partners in Kenya to refine and improve interventions to better serve adolescent girls and young women who are at risk of HIV infection.

### 2.0 Study objectives

The overall goal of this study is to identify multi-level risk factors associated with PrEP uptake and adherence outcomes among AGYW, using a combination of de-identified programmatic data from the DREAMS Initiative and supplemental contextual information provided through interviews with a sample of DREAMS participants. Note that, as per PATH REC staff guidance, the analysis of de-identified DREAMS programmatic data is not included as a component of this protocol since it has qualified for a non-research determination.

The primary objectives of the study are as follows:

1. To measure PrEP adherence via self-report, return visits, and biomarkers and evaluate concordance between these measures.
2. To identify motivations for taking PrEP, perceived challenges with adherence to the PrEP regimen and reasons for discontinuing PrEP.
3. To explore the experience of care as it relates to receiving PrEP via community safe spaces.
4. To identify individual, familial and community level factors among AGYW that may impact persistence and adherence to the PrEP regimen.
5. To identify challenges to, and factors that facilitate, PrEP adherence from the perspective of the DREAMS mentors.

Secondary objectives include the following:

1. To identify current level of HIV risk and perceptions of HIV risk among AGYW enrolled in DREAMS and taking PrEP.
2. To explore the attitudes of AGYW as peers regarding HIV risk and PrEP for HIV prevention.
3. To explore the attitudes and practices of health care providers with respect to AGYW reproductive health services and HIV prevention (with focus on PrEP).

### 3.0 Study population, study design, and sample size

**Study population**

Young women and adolescent girls ages 18-24 years who are taking PrEP and enrolled for between 2 and 9 months will be randomly identified from DREAMS programmatic data in select wards of Kisumu and Homabay counties, located in Western Kenya. Under the DREAMS program, AGYW are assessed for PrEP eligibility and must be HIV- with at least one HIV risk factor identified. PrEP eligibility is re-assessed on a quarterly basis.

The status of PrEP enrollment for the two counties as of November 2017 is shown in Table 2, although since the DREAMS project is on-going, the number of AGYWs on PrEP will increase as additional AGYW are added prior to participant recruitment. The selection of specific wards is to be determined and will be driven by the number of potentially eligible participants (PrEP enrollees) by ward. We expect to recruit from approximately 10 wards, across both counties.

**Table 2: Number enrolled on PrEP by ward, Homabay and Kisumu counties (Nov. 2017)**

| **Homabay County** | **Ward** | **Freq.** | **Percent** |
| --- | --- | --- | --- |
|  | East Gem | 97 | 13.76 |
|  | Rusinga Island | 55 | 7.8 |
|  | North Kabuoch | 46 | 6.52 |
|  | Homa Bay West | 41 | 5.82 |
|  | West Gem | 39 | 5.53 |
|  | Homa Bay Arujo | 32 | 4.54 |
|  | Kendu Bay Town | 31 | 4.4 |
|  | Central | 28 | 3.97 |
|  | Ruma Kaksingri East | 28 | 3.97 |
|  | Kwabwai | 27 | 3.83 |
|  | Homa Bay Central | 25 | 3.55 |
|  | North Karachuonyo | 24 | 3.4 |
|  | Kasgunga | 23 | 3.26 |
|  | Kanyaluo | 20 | 2.84 |
|  | Kibiri | 20 | 2.84 |
|  | Kanyikela | 19 | 2.7 |
|  | Homa Bay East | 18 | 2.55 |
|  | Kochia | 18 | 2.55 |
|  | Kanyamwa Kologi | 17 | 2.41 |
|  | Kanyamwa Kosewe | 16 | 2.27 |
|  | Kagan | 14 | 1.99 |
|  | Kanyadoto | 14 | 1.99 |
|  | West Karachuonyo | 13 | 1.84 |
|  | Wangchieng | 11 | 1.56 |
|  | Gwassi South | 9 | 1.28 |
|  | Gwassi North | 8 | 1.13 |
|  | Kabuoch South/Pala | 8 | 1.13 |
|  | Mfangano Island | 4 | 0.57 |
| **Total Homabay** |  | **705** | **100** |
| **Kisumu County** | **Ward** | **Freq.** | **Percent** |
|  | Kajulu | 179 | 27.62 |
|  | Kolwa East | 150 | 23.15 |
|  | Nyalenda 'A' | 141 | 21.76 |
|  | Manyatta 'B' | 92 | 14.2 |
|  | Kolwa Central | 86 | 13.27 |
| **Total Kisumu** |  | **648** | **100** |

**Study design**

The proposed study is a prospective mixed methods study involving up to two biomarker samples and two interviews to measure PrEP adherence and identify factors affecting adherence among AGYW who are enrolled in DREAMS and receiving PrEP for HIV prevention. The inclusion of biomarker data for a share of the DREAMS participants who are enrolled in PrEP will provide a valid measure of DREAMS adherence (the dependent variable) for the analysis of associated or contributing factors drawn from the DREAMS database.

AGYW enrolled in the DREAMS/PrEP program for between 2 and 9 months will be recruited for participation in one-on-one interviews and the biomarker sampling. The first time point for interviews may therefore vary anywhere from 2 to 9 months into the PrEP program for each participant, which will allow for a range of program exposures among study participants. Interviews will be repeated three to four months after the first interview/DBS, and if the participant has continued with PrEP, a second DBS sample will be collected. Conducting repeat interviews and DBS will allow an assessment of how events in an AGYW’s life situation may impact her PrEP adherence and persistence over time.

**Figure 2: Sample of AGYW study participants**

**Sample at Time 1** **Sample at Time 2**

Longitudinal sample

Longitudinal sample

We will supplement and enhance our understanding of factors critical to PrEP adherence through qualitative data collection. Specifically, we will conduct two focus groups with AGYWs who are 18+ years old (AGYW peers) but not enrolled on PrEP, two focus groups with DREAMS health care providers and two focus groups with DREAMS mentors who coordinate the DREAMS Safe Space activities. The AGYW peer focus groups are intended to explore the influence of peers on AGYWs’ PrEP enrolment and adherence. We target AGYW who are not themselves enrolled on PrEP in order to better understand the social climate in the communities where our participants reside. Understanding the social environment is critical for studies involving AGYW because of the strong influence these social spheres have over AGYW’s health and behavior.^[[46]](#endnote-46)^ Focus group participants will be drawn from the same wards as the AGYW who participate in interviews and biomarker sampling.

Finally, we will conduct in-depth interviews (IDIs) with a subsample of 16 AGYW 18+ years who are continuing with PrEP and in-depth interviews with 16 AGYWs 18+ years who have discontinued PrEP entirely. These interviews are for the purposes of illuminating in a qualitative fashion some of the underlying behavior rationales affecting adherence as well as the underlying reasons *why* AGYWs discontinue PrEP use after enrollment. The samples of AGYW for the IDIs will be randomly drawn from among those participating in the quantitative survey (a subsample of respondents).

The outcome measure or study endpoint will be PrEP adherence as measured by the biomarker level of tenofovir. Multivariate regression analysis will determine whether differences in adherence are associated with any particular contextual co-factors. Specifically, we hypothesize that the following measures will be positively associated with PrEP adherence:

- AGYW perceived HIV risk and perceived efficacy of PrEP
- Internal and perceived HIV stigma
- Level of personal empowerment
- Perceived social support of family, friends (MODS survey)
- Trust, confidence in provider and mentor
- Level and nature of involvement in DREAMS activities
- Regularity of PrEP provision at Safe Spaces
- Receptivity of provider to AGYW use of PrEP
- Community support for DREAMS, PrEP
- Lower depression scores (PHQ-9)
- Lower alcohol use (AUDIT)
- Lower intimate partner violence (PVS/HITS)
- Higher food security (USDA/WHO)

**Sample size**

The sample size of AGYW who will participate in the interviews and provide biomarker samples at Time 1 is 350. Interviews will be repeated after 3 to 4 months for all participants, and biomarkers collected again for those who continue in the PrEP program. Assuming a continuation rate of 40% (and 10% loss to follow up), we expect to collect 175 repeated DBS samples, for a total of 525 samples.

PrEP Adherence at each time point will be primarily measured by the percentage of AGYW whose PrEP drug levels in dried blood spots reached a certain threshold reflecting the exposure to PrEP drug in the recent past. Specifically, PrEP adherence will be measured by the percentage of AGYW whose tenofovir-diphosphate drug levels in DBS exceeds 30f/molx106, which is consistent with an average of at least four PrEP doses per week.

Assuming a discontinuation/ loss to follow-up rate of 50%, and a two-level factor (potentially associated with PrEP adherence), this sample size will allow us to detect cross-sectionally at the first time point a minimum absolute difference of 15% in the PrEP adherence rates between the levels of the factor at 80% power given a type I error rate of 5% and a two-sided test (Table 3).

**Table 3.** Sample size and power considerations for AGYW interview and DBS samples

| **Absolute difference in adherence rates** | **Sample size needed to measure the absolute difference in the adherence rates** | |
| --- | --- | --- |
|  | **80% power** | **90% power** |
| 25% | 136 | 178 |
| 20% | 206 | 270 |
| 15% | 350 | 462 |
| 10% | 752 | 992 |

Table 4 presents the two-sided 95% confidence intervals for the true PrEP adherence rates at the two time points (for DBS sample and interviews) for a discontinuation/loss to follow-up rate of 30% or 50%. For example, there is a 95% chance that the true PrEP adherence rate at Time point 1 is in the range (44.76%, 55.24%) if the observed adherence rate is 50% at this time point. Moreover, assuming a discontinuation/loss to follow-up rate of 50%, there is a 95% chance that the true PrEP adherence rate at Time point 2 will lie in (23.21%, 36.79%) if the observed adherence rate is 30% at Time point 2. Furthermore, the 95% confidence intervals for the absolute difference in the PrEP adherence rates between the two visits are also shown in Table 4. For example, the absolute difference in the true PrEP adherence rates between Time point 1 and Time point 2 will be in the range (11.85%, 28.15%) with a probability of 0.95 if the observed adherence rates are 50% and 30% at Time points 1 and 2, respectively, assuming a discontinuation/loss to follow-up rate of 50% and a correlation of 0.1 between the adherence measures at the two time points.

**Table 4:** 95% confidence intervals for the true PrEP adherences given by possibly observed PrEP adherence rates at the two time points.

| **95% CI for true the PrEP adherence based on the observed PrEP adherence rates by visit** | | | | |
| --- | --- | --- | --- | --- |
| **Time point 1** | | **Time point 2** | | |
| **Observed PrEP adherence rate** | **95% CI for the true PrEP adherence rate** | **Observed PrEP adherence rate** | **95% CI for True PrEP adherence rate** | |
|  |  |  | **30% discontinuation/ loss to follow-up** | **50% discontinuation/ loss to follow-up** |
| 30% (Low) | (25.2%, 34.8%) | 10% | (6.24%, 13.76%) | (5.56%, 14.44%) |
|  |  | 20% | (14.99%, 25.01%) | (14.07%, 25.93%) |
| 50% (Medium) | (44.76%, 55.24%) | 30% | (24.26%, 35.74%) | (23.21%, 36.79%) |
|  |  | 40% | (33.87%, 46.13%) | (32.74%, 47.26%) |
| 70% (High) | (65.2%, 74.8%) | 50% | (43.74%, 56.26%) | (42.59%, 57.41%) |
|  |  | 60% | (53.87%, 66.13%) | (52.74%, 67.26%) |
| **95% CI for the absolute difference in the true PrEP adherence rates between the two visits** | | | | |
| **Observed PrEP adherence ratet Visit 1** | **Observed PrEP adherence rate at Visit 2** | | **30% discontinuation/ loss to follow-up** | **50% discontinuation/ loss to follow-up** |
| **CORR=0.1** | | | | |
| 30% (Low) | 10% | | (14.21%, 25.79%) | (13.79%, 26.21%) |
|  | 20% | | (3.42%, 16.58%) | (2.76%, 17.24%) |
| 50% (Medium) | 30% | | (12.63%, 27.37%) | (11.85%, 28.15%) |
|  | 40% | | (2.34%, 17.66%) | (1.48%, 18.52%) |
| 70% (High) | 50% | | (12.5%, 27.5%) | (11.58%, 28.42%) |
|  | 60% | | (2.6%, 17.6%) | (1.71%, 18.29%) |
| **CORR=-0.1** | | | | |
| 30% (Low) | 10% | | (13.61%, 26.39%) | (13.14%, 26.86%) |
|  | 20% | | (2.72%, 17.28%) | (2.01%, 17.99%) |
| 50% (Medium) | 30% | | (11.85%, 28.15%) | (11.02%, 28.98%) |
|  | 40% | | (1.54%, 18.46%) | (0.63%, 19.37%) |
| 70% (High) | 50% | | (11.74%, 28.26%) | (10.78%, 29.22%) |
|  | 60% | | (1.84%, 18.16%) | (0.91%, 19.09%) |

Note: CORR denotes the correlation between the two adherence measures.

For the qualitative component, we plan to conduct two focus groups with AGYW peers who are not participating in the PrEP program, two focus groups with Safe Space mentors, and two with health providers, and each group will have 8 to 10 participants. Hence the estimated total sample size is 48 – 60 focus group participants.

For the qualitative interviews with PrEP continuers and discontinuers, a sample size of 16 AGYWs of each type, is considered sufficient to achieve saturation on the themes of interest.^[[47]](#endnote-47)^ The PrEP continuers will be randomly selected to participate in in-depth interviews from among the survey respondents who continue with PrEP, while the discontinuers will be randomly selected from among survey respondents who have discontinued PrEP.

**Data analysis**

AGYW will be considered adherent if their tenofovir-diphosphate drug levels in DBS exceed 30f/molx106, which is consistent with an average of at least four PrEP doses per week. Thus, adherence measured by dry blood spot is a dichotomous measure (Yes, if drug concentration level exceeds 30f/molx10^6 and No otherwise). We will also examine adherence based on the AGYW self-report and for analysis of concordance between adherence measures (e.g. self-report and drug concentration level-based measures), we will calculate Cohen's kappa coefficient as well as sensitivity, specificity and positive predictive values, which are commonly used to assess the degree of agreement between two binary rates.^[[48]](#endnote-48)^ ^[[49]](#endnote-49)^

Data analysis will be done by study team staff who have both qualitative and quantitative analysis experience and skills. Data analysis of quantitative data will be conducted using SAS, STATA or R. Descriptive statistical analysis including frequencies of responses and distribution will be used to summarize factors influencing PrEP uptake and adherence. Chi-square tests will be employed to make comparisons between groups, such as married and unmarried. Multivariate regression analysis will determine whether differences in adherence rates are associated with any particular contextual co-factors, such as the experience of intimate partner violence, levels of social support, perception of HIV stigma, self-efficacy, depression, alcohol use, etc.

Focus groups and in-depth interviews will be translated, transcribed, and analyzed thematically using qualitative software, such as Atlas.ti. Focus group and in-depth interview analysis will explore factors that facilitate or are barriers to PrEP uptake and adherence for AGYW from the perspective of peers, mentors and health workers (focus groups) and will explore these factors more in-depth in the qualitative interviews with young women who continuer or discontinuer PrEP.

### 4.0 Study procedures

The study procedures involve two one-on-one semi-structured interviews conducted by study staff with the AGYW. These interviews will take not more than 1 hour. The interviews will take place at private spaces in the DREAMS program ‘safe spaces’ or, if preferred by participants, may alternatively be conducted at other community locations, places of residence or the workplace as convenient and desired by the participant.

We will conduct one follow-up visit with each AGYW after an interval of 3 to 4 months. At this visit study staff will conduct a second interview. If the AGYW is continuing with PrEP, questions will focus on adherence and factors that impact adherence. If the AGYW has discontinued PrEP, questions will focus on reasons for discontinuation. Illustrative lines of inquiry for both interviews are outlined below.

We will collect a dried blood spot (DBS) sample from each AGYW who is continuing in the PrEP program at two points in time, separated by an interval of 3 to 4 months. PrEP discontinuation is defined as 60 or more days since due for a PrEP refill, and AGYW reports that she is not currently taking PrEP. Those who have discontinued will participate in the interview only.

A trained HTS (HIV testing services) worker -- trained in research ethics, blood sampling via fingerstick, and interviewing techniques -- will be employed as study staff to take the samples and conduct the interviews.

In addition to the DBS collected from study participants, we will recruit, consent, and collect a DBS from up to 10 HIV+ AGYW who are on ART (TDF) and virally-suppressed to serve as positive controls for the purpose of validating the DBS storage and testing procedures. Because these individuals are virally suppressed from their ART, their DBS test results will show adherent tenofovir levels, assuming an accurate test assay and effective DBS collection, storage, and transfer procedures. Other than the DBS, their age, their length of time on ARTs, and duration of viral suppression (if known), no additional information from these positive controls will be collected. To clarify, these participants are not contributing toward the achievement of the study objectives per se, nor providing data for the analysis, but are critical to validate that the DBS testing results obtained from study participants are accurate measures of PrEP adherence. PATH has a project, called Afya Ziwani, that provides services to HIV+ individuals in Kisumu and Nyamira counties and therefore is able to identify women on ART who, if willing, could serve as positive controls.

*Quantification of PrEP Adherence:* We will measure tenofovir diphosphate (TFV-DP) in finger-prick whole blood dried blood spots. Dried blood spots (DBS) have been demonstrated to be a suitable measure for TFV-DP, as a long-term measure of TDF and PrEP adherence. Approximately 2.5 ml of blood is required for the dried blood spot card.

All drug measurements will be performed at the Program for HIV Prevention and Treatment (PHPT) laboratory at Chiang Mai University (ISO:15189 certified), pharmacology laboratory established by Dr. Tim Cressey in 2003. The Chiang Mai University laboratory is approved by the Division of AIDS (DAIDS) to perform antiretroviral drug level measurements by liquid chromatography dual mass spectrometry (LC-MS/MS), and is the reference laboratory for IMPAACT and DAIDS studies in Thailand. In accordance with DAIDS polices, the lab participates in the Clinical Pharmacology Quality Assurance and Quality Control Program (CPQA).[^63^](#_ENREF_63) The CPQA program includes regular proficiency testing, external review of SOPs/validation reports, and audits. Drug assays used by the lab are validated based on the US FDA ‘Bioanalytical Method Validation’ guidelines.

This research approach was developed in Peter Anderson’s lab at the University of Colorado, and the seminal paper is the following:  Castillo-Mancilla JR, et al. "Tenofovir, emtirictabine, and tenofovir diphosphate in dried blood spots for determining recent and cumulative drug exposure.” In this paper, the authors describe the protocol and validation study for using DBS for the measurement of cumulative TFV-DP. In 2017, Dr. Tim Cressey and his team spent 2 months as Visiting Scientists in Dr. Anderson’s lab learning the protocol for testing TFV-DP from DBS. The Cressey lab is now using the same protocol for the DBS testing using dual liquid chromatography mass spectrometry that was pioneered in the Anderson lab.  The Cressey lab is currently testing DBS samples in the TARGET Study (PI:  Paul Drain, who is a co-investigator on the current study), and the quality of results has been excellent.

This lab and the lab in Colorado are the only labs that are measuring tenofovir diphosphate (TFV-DP) in finger-prick whole blood dried blood spots.

*Storage of Samples*: All of the DBS samples will be stored in a local -20 C freezer. The samples will be shipped frozen to the PHPT laboratory in Chiang Mai, Thailand. Once received, the samples will again be placed in a -20 C freezer until the time of testing. The study will develop SOPs for study staff to detail DBS sampling procedures, storage, and transport logistics.

IDIs with PrEP continuers and discontinuers (a subset of survey respondents) will be scheduled following the second quantitative interview, based on timing that is convenient to the participant. The IDIs will last no more than one hour.

Focus group discussions will be conducted at Safe Spaces or other appropriate venue providing participants with adequate privacy. The study will employ a trained facilitator to conduct the focus group discussions. They will be audio-recorded.

### 5.0 Eligibility criteria

Note that DREAMS participants have previously consented to be contacted about possible participation in future studies of this type. The legal age of consent in Kenya is 18 years.

**Inclusion criteria:**

AGYW will be eligible for participation in the research study (interviews and/or DBS sampling) if they meet the following criteria:

- Females enrolled or re-enrolled (after a program pause in 2018) on PrEP for 2 to 9 months through the DREAMS initiative
- Consented to be contacted for future studies on the DREAMS program consent form.
- Age 18-24 years
- Residing in select wards of Kisumu or Homabay county
- Provide informed consent to participate in the study

For the IDIs with PrEP continuers, AGYW will be eligible if they meet the following additional criterion:

- Continued with regular PrEP refill visits for at least five months

For the IDIs with PrEP discontinuers, AGYW will be eligible if they meet the following criteria:

- Discontinued PrEP as demonstrated by having missed at least two refill visits (> 60 days overdue) by the time of the 2^nd^ interview and AGYW reports that she is not currently taking PrEP.

AGYW peers will be eligible for participation in the focus group discussions if they meet the following criteria:

- Participating in the DREAMS program but not on PrEP
- Females ages 18+ years
- Residing in select wards of Kisumu or Homabay county
- Provide informed consent to participate in the study

DREAMS Mentors will be eligible for participation in the research study if they meet the following criteria:

- Females ages 18+ years
- Identified by the DREAMS staff as a DREAMS Initiative mentor who coordinates Safe Space activities in the target study wards.
- Provide informed consent to participate in the study

Health care providers will be eligible for participation in the research study if they meet the following criteria:

- Ages 18+ years
- Identified via DREAMS staff as a health provider who provides PrEP services for AGYW in the target study wards
- Received training in PrEP, as per DREAMS program records
- Provide informed consent to participate in the study

AGYW serving as positive controls for DBS collection, storage, and testing procedures must meet the following criteria:

- Female
- Ages 18+ years
- HIV+ Afya Ziwani program participant
- Residing in Kisumu or Nyamira county
- On an ART regimen that includes Tenofovir Disoproxil Fumarate (TDF)
- Virally suppressed, as demonstrated by whole blood plasma testing within the past 4 weeks AND penultimate test also demonstrating viral suppression
- Provide informed consent to participate in the study

**Exclusion Criteria:**

For AGYW, peers, mentor and health care providers, individuals will be excluded from participation in the study based on the following:

- Speaking a language other than English, Dholuo, or Kiswahili
- Unable to provide informed consent due to social or mental challenges

### 6.0 Recruitment

*Recruitment for interviews and DBS*

AGYWs enrolled in the DREAMS Initiative attend safe spaces at least once a month for DREAMS program activities. As part of participating in DREAMS, AGYWs were asked if they were willing to be contacted for future studies and that they would be given the opportunity to decide if they wanted to participate or not.

A random sample of 350 AGYWs from the selected wards who have been enrolled in PrEP for 2 to 9 months, and who indicated willingness to be contacted for future studies, will be generated using a computer-generated random number list, from the DREAMS data for the selected wards. DREAMS participants are identified in the DREAMS data by a unique ID number. The DREAMS team in Kenya (PATH staff who manage the DREAMS project monitoring and evaluation) will link the ID numbers in the drawn sample to the contact information for potential study participants that is recorded/stored separately by the DREAMS project. A study staff will contact the AGYW who have indicated a willingness to be contacted about future studies via phone or at the Safe Space to introduce the study using an approved recruitment script.

The recruitment of participants will take place at monthly PrEP refill visits – designated meeting times when PrEP enrollees come to the Safe Spaces to receive their PrEP refills from providers. DBS sampling will take place at that time, for those who consent to participate. We will make appointments for the interviews to take place after the DREAMS Safe Space meeting, or within the next few days, whichever is convenient for the participant.

*Recruitment for focus group discussions*

For the focus groups with AGYW peers, we will randomly select and recruit 20 AGYW who are participating in DREAMS in the select study wards, but who are not enrolled on PrEP. Focus group participants will be drawn from AGYW attending Safe Space activities, and invited to participate using a standardized recruitment script.

For the focus groups with mentors, we will randomly select potential participants from among those mentors operating in the same wards as the AGYW study participants. Study staff will contact mentors using the contact information available on record and invite them to participate in the study using a standardized recruitment script. Mentors are aware that they may be contacted for research studies and other related activities due to their participation in DREAMS, and as with any participant, may voluntarily choose to participate in the study or not.

Health care providers who are assigned to the DREAMS program Safe Spaces in the same wards as the AGYW study participants, and who have received training in PrEP will be randomly selected and recruited. These providers have responsibility for providing routine PrEP refills and assessments for DREAMS participants at the Safe Space. A study staff will approach health care providers during these visits or at their place of work to ask if they are willing to participate in the study following an approved recruitment script.

*Recruitment for in-depth interviews*

Participants who meet the additional eligibility criteria for either type of IDI (continuing PrEP or discontinuing PrEP) will be randomly selected from among those participating in the second interviews. In-depth interviews will be audio-recorded, conducted at a place of convenience to the respondent, and lasting no more than one hour.

*Recruitment of positive controls*

The Afya Ziwani team collects data on HIV+ AGYW who participate in their program. Based on their program records, the Afya Ziwani team will identify a convenience sample of potential positive controls who meet the eligibility criteria (female 18+ years old, living in Kisumu or Nyamira county, taking tenofovir for ART, virally suppressed as per testing within the past month, and penultimate viral load test demonstrating suppression). An Afya Ziwani staff person will contact the individual about their willingness to be contacted about future studies via phone or at the Safe Space. S/he will introduce the study and their specific role in the study using an approved recruitment script. The role of the Afya Ziwani project staff (who are not research staff) ends with identification and preliminary recruitment of potential participants whose medical records suggest they fit the eligibility criteria. Once a potential participant agrees to meet with a PATH research staff person, all study procedures are conducted by study staff, including informed consent, verification of eligibility and DBS sampling.

For those who are interested, an appointment will be made to assess eligibility and conduct the informed consent. Note that the consent form includes release of the patient information relevant for eligibility assessment (type of ART, viral load at most recent test, date of most recent test, viral load at penultimate test); study staff will be blinded to this patient information unless the participant consents to share it. For individuals who are eligible and consent to participate, a DBS will be collected. Once the DBS is collected, their participation in the study ends.

Recruitment and participant engagement procedures will be adapted to the COVID context. Specifically, the procedures outlined below will be followed to ensure the safety of participants and research staff:

- We will comply with country and county public health authorities and government requirements. Through Afya Ziwani, the study team will meet with members of sub-County and County Health Management to discuss study plans including plans to adhere to all COVID-19 protocols.
- We will ensure that water and soap for handwashing and/or hand sanitizers are available in all locations where individuals will meet the study team. In addition, we will provide participants in this study with a face mask.
- DBS samples will be collected at a central place. The study team will cater for transport costs for both the participant and the study team. Transport costs incurred by participants to come to central locations for purposes of the study will be reimbursed as per PATH Kenya guidelines. We will ensure that individuals follow the recommended guidelines for physical distancing when using public means to travel to these locations.
- Data collection will be done through finger prick for purposes of DBS collection. We will ensure that the study team member performing the DBS procedures and the participant wear a face mask throughout the contact period.
- Further, we will ensure the study team member and the participant maintain physical distance of at least 2 meters except while pricking the participant’s finger and drawing her blood.
- DBS collection will be done in an open and secluded space, away from buildings and with one participant at a time.
- The study team will carry an infrared thermometer for checking their own and study participants’ temperature.
- Participants and the study team will be informed not to come for any in-person data collection activity if they are not feeling well.

### 7.0 Illustrative lines of inquiry

**AGYW participant**:

| **Topic** | **1^st^ interview** | **2^nd^ interview** |
| --- | --- | --- |
| Principal rationale or motivation for participating in the DREAMS program | X |  |
| Principal rationale or motivation for initiating PrEP | X |  |
| Experience of care, receiving PrEP via Safe Spaces | X |  |
| Self-reported adherence to PrEP regimen | X | X |
| Perceived challenges with PrEP adherence | X | X |
| Reasons for discontinuing PrEP |  | X |
| Level of social support (including partner, family and community) for PrEP | X | X |
| Current level of HIV risk and perception of HIV risk | X | X |
| Perceived HIV stigma | X | X |
| Perceived efficacy of PrEP | X | X |
| Degree of self-efficacy/personal empowerment | X |  |
| Indicators for depression/mental health | X | X |
| Alcohol use | X | X |
| Experience of violence | X | X |
| Food security | X | X |

**AGYW focus groups**:

- Level of engagement and support in/for DREAMS Initiative
- Beliefs about factors that increase or decrease risk of HIV acquisition
- Stigma associated with HIV
- Perceptions, attitudes and beliefs about PrEP use and AGYW who take PrEP

**DREAMS mentor focus groups**:

- Awareness of factors that impact AGYWs’ enrollment on PrEP, including social and cultural factors that facilitate or impede PrEP use
- Awareness of factors that impact PrEP adherence.
- Perceptions about benefits and challenges of providing PrEP at community venues compared to health facilities

**Health care provider** focus groups:

- Attitudes, beliefs regarding provision of reproductive health services to AGYW
- Perception of level of community support for DREAMS, PrEP
- Perceptions about benefits and challenges of providing PrEP at community venues compared to health facilities
- Knowledge of social and cultural factors that facilitate or impede PrEP use.

**AGYW PrEP continuing participant:**

- Assessment of satisfaction with DREAMS program activities
- Deeper exploration of principal rationale or motivation for initiating PrEP
- Deeper exploration of level of social/community support for PrEP
- Deeper exploration of perceived challenges with PrEP adherence

**AGYW PrEP discontinuing participant**:

- Assessment of satisfaction with DREAMS program activities
- Exploration of rationales for discontinuing PrEP
- Deeper exploration of perceived challenges with PrEP adherence
- Perceived level of social/community support during PrEP use
- Deeper exploration of current level of HIV risk and perception of HIV risk

### 8.0 Consent process

The consent process for AGYW will take place in a private setting at the DREAMS program safe spaces for AGYW. If preferred by participants, the consent process may also be conducted at other community locations, places of residence or place of work.

Study staff will explain all aspects of the study to the potential participants, emphasize that participation in this study is voluntary, and that the health care services that a participant receives will not be affected by her decision to participate or not in the study. Potential participants will be given the opportunity to read the consent themselves and to ask questions of the study staff.

If a potential participant is unable to read the consent and requires it to be read to her, a literate, impartial witness will serve as a consent witness to ensure that the participant understands the study information, including risks and benefits. In addition to being literate, the witness must not be a member of the research team or a PATH staff person. A Safe Space mentor or health care provider are examples of suitable informed consent witnesses. Note that the witness would exit the room following the consent process and therefore would not be present during the interview.

Once all the potential participant’s questions have been answered satisfactorily, potential participants will be given the opportunity to voluntarily indicate if they wish to participate in the study. If an individual agrees to participate, she will be asked to sign, or give her initials if she is unable to sign. Participants will sign two identical consents and will be given a copy of one of the consents to take home.

The consent process for positive controls is the same as for AGYW study participants, though the consent form itself is tailored to their role.

The consent process for the focus group discussions may be conducted individually or in a group setting, given the low risk of the activity. Basic demographic information (age, education, marital status, length of involvement in DREAMS, job title (providers)) will be collected from participants for the purposes of defining the focus group population. Personal identifiers will not be collected. Given the anonymity of their participation in the FGD process and low risk of the activity, consenting for focus group discussions will be oral, with the study staff signing to verify that the consent process was followed. A consent log will be created to track the consent status of each individual recruited.

For the in-depth interviews and focus group discussions, participants who are unwilling to be audio-recorded will not participate.

Study staff responsible for obtaining consent will be trained in human subjects protection, including the importance of not coercing or influencing individuals’ decision to participate in the study. A private setting will be used for the consent process in order to protect the privacy and confidentiality of potential participants during the process.

### 9.0 Risks

The risks for participants in this study due to the study procedures (interview) are anticipated to be minimal. Because our target population is AGYW who are enrolled in the DREAMS program, they are more accustomed than the average Kenyan woman to discussing sensitive topics. As an example, AGYW who are taking PrEP are asked on a quarterly basis about their risk factors for HIV infection, including questions that concern transactional sex, the number of sexual partners, gender-based violence, etc. The risks associated with participating in the current study may include the following:

Psychological risk: Participants may feel embarrassment or discomfort discussing sensitive topics such as sexual behavior, HIV services used, etc.

Social risk: There is a risk of a breach of confidentiality, which could put an individual at risk if someone were to find out sensitive and confidential information about the AGYW. For the positive controls, a breach of confidentiality could reveal her status as HIV+. Similarly, if there were a breach of confidentiality, a health care provider could be put at risk with her/her employer or community if his/her responses were viewed negatively.

In the focus group discussions, the study will ask participants not to talk with others outside the study about who was in the group or what was discussed. However, there is the risk that a participant may breach this. Also note that, by participating in an FGD, the individual is self-disclosing to other participants that they are involved in the DREAMS program, either as a participant (peer), provider, or Safe Space mentor.

Physical risks (blood sample): The participant will likely feel some pain when the staff conducts the fingerstick to take the blood sample. Other than this brief pain, the discomfort of the finger prick is minimal. This pain is comparable for tests for HIV infection, which are conducted on a quarterly basis with all PrEP participants. In about one out of 10 cases, people have a small amount of bleeding under the skin or a bruise. For some people they may have a small scar for a few weeks. The risk for localized infection from the fingerstick is less than 1 in 1,000.

### 10.0 Mitigation of risks

Efforts will be made by study staff to mitigate risks of study participation described above as follows:

A private setting will be used for the all participant interviews. All participants (AGYW, peer, mentor and health care providers) will be informed that they do not have to answer any question they do not wish to answer, and that they are free to withdraw from the study at any time. Interviewers will be trained to remind participants of this during the interview if a participant is visibly embarrassed or uncomfortable by a question.

For the positive controls (who are HIV+), all interactions with potential participants will be conducted in a private setting to minimize the risk of any breach of confidentiality. In addition, participants will be given the option to decline to take a copy of the ICF so as to reduce possible exposure of her HIV+ status at a later time.

In the focus group discussions, the study will ask participants not to talk with others outside the study about who was in the group or what was discussed. Participants will also be asked to not use names during the discussions e.g., when talking about young women or referencing each other. The study will redact names in the transcripts that may be inadvertently mentioned during the discussions.

To lessen risks of bruising and infection from the fingerstick blood sample, the study will employ experienced HTS workers as study staff to take the blood samples. These staff are experienced with handling blood products, and will have additional training to take the blood samples, consistent with sampling and testing requirements for Tenofovir.

AGYW participants who do not want to have their blood sampled for TDV/FTC testing will be counseled that choosing not to have their blood sampled and/or not participating in the interview will not affect their participation in the DREAMS program or their enrollment on PrEP.

All efforts will be made to maintain confidentiality and data security. Study staff will participate in training for study confidentiality, including conducting interviews and focus groups in private settings, securing data on password protected devices, keeping paper forms and tape recordings in locked cabinets/rooms, and maintaining all information collected by the study as confidential, not to be shared with individuals who are not part of the research team. Consent witness will also be trained regarding ethics of research consent, with particular emphasis on protecting the confidentiality and privacy of participants.

### 11.0 Benefits

Participants of the study will receive no direct benefit. However, DREAMS participants, including those who participate in the study, may benefit in terms of improvements in the way PATH implements the DREAMS program and the provision of PrEP services, based on the insights they provide.

We expect the societal benefits of the study may be to women and girls, and their communities in Kenya and potentially beyond by furthering knowledge about how to increase adherence to PrEP for this population and hence decrease the HIV burden. Input from DREAMS program participants – AGYW, mentors and providers – may help to improve the services received through DREAMS.

### 12.0 Study and safety monitoring

Study staff will make routine, biweekly visits to the field during data collection to meet with interviewers and discuss study implementation, with a focus on the informed consent process, preservation of confidentiality, data management and security, challenges with contacting participants or other recruitment issues, challenges with data collection, etc.

In addition, to ensure that study staff are implementing the study according to the protocol, an audit will be conducted in which a sample of participants who have been interviewed to date are contacted to confirm their involvement, and ensure that informed consent procedures were followed. Participants will be selected at random, and interviewers will not know in advance which participants will be contacted.

### 13.0 Managing and reporting adverse events

An adverse event (AE) is any untoward or undesirable event experienced by a participant regardless of whether the event is expected or related to the participant’s involvement in the research. Given the nature of the study intervention (an interview and DBS), we do not anticipate any serious adverse events in this study aside from the risks described in previous sections. See also the next section.

### 14.0 Managing and reporting unanticipated problems or protocol deviations

In this study, an unanticipated problem is any incident, experience or outcome (physical, psychological, economic or social) that potentially places the participants or others at greater risk for harm.

Participants will be provided with contact information for staff (in the informed consent) and reminded that they should contact the staff in the event of any unanticipated problems arising from their experience as a study participant.

Staff are required to report any unanticipated problems or deviations to protocol that occur during the study to the Study PI within 24 hours of learning about issue. Within 72 hours, the Study PI will notify involved appropriate Ethics Committees in accordance with their reporting requirements.

### 15.0 Confidentiality and data management

All study staff will participate in training for study confidentiality as follows: Study staff will maintain all information collected by the study as confidential. Only study staff will have access to data, tape recordings, and personal information that identifies study participants. Only study investigators will have passwords to access computers and keys for files where study data (including tape recordings) are stored. Only study identification numbers will be used to label study records, tape recordings, and transcripts. Any identifying information will be deleted/redacted from transcripts.

We will store the tape recordings until they are transcribed into computer records. We will erase tape recordings as soon as possible after they are no longer needed.

We will only label blood samples with a study identification number and no other identifying information. We will store samples securely and only allow relevant study staff to access samples. Samples will be stored for up to five years after study activities have been completed and then be destroyed. Samples will only be used for this study to test for PrEP medication levels. The samples will not be tested for HIV or any other tests in this or any future studies. Linking documents connecting the participant’s name and other identifying information to the study identification number will be maintained for quality control (auditing) and any necessary follow-up (missing data, etc.). Linking documents and other personal information will be destroyed as soon as possible within five years after study activities have been completed e.g., after all study data has been verified and cleaned and/or necessary follow-up has been conducted.

Study reports and presentations will not contain any personal identifying information of any study participant.

Trained study staff will collect semi-structured data from study participants electronically using mobile phones or tablets, using Open Data Kit or similar data collection software. Data will be uploaded to a server immediately post-interview or as soon as connectivity can be established if the network is poor. Phones and/or tablets will be password protected and personal identifying data will not be entered. Paper questionnaires may be used in the event of technological challenges and/or poor connectivity. These documents will only be labeled with the study identification number.

The in-depth interviews will be audio recorded. ID numbers will be used to identify participants and names will not be collected on the audio recording. Similarly, the recordings will be transcribed to password protected computers, and the transcripts will contain only the study ID number.

Data collected in the study will not be disclosed to any health officials, will not be entered into the medical record of the participant, nor reported to health center.

In accordance with funder’s requirements, de-identified data will be made available in a data repository. Study records will be retained for 5 years after the end of the study and then destroyed.

### 16.0 Study costs

Study participants will not need to pay to participate in the study. Participants will not incur cost to participate in the study beyond what they would normally incur when they present for a PrEP visit.

### 17.0 Care for injury

Since this study involves only the administration of a questionnaire, no injuries are expected as a result from the activities in this study.

### 18.0 Compensation

Participants will receive a small token of appreciation, such as refreshments, to thank them for their participation. The token will be no more than USD $3 in value. This is considered a nominal amount and consistent with research study practices in the area.

### 19.0 Investigator responsibilities

List the investigators’ responsibilities in conducting and managing this study, e.g., training, documentation, oversight, reporting.

Jane Cover, Seattle-based PI:

- Overall responsibility for study design and implementation
- Analysis and synthesis of data
- Liaising with PATH REC and study sponsor

Christopher Obong’o, Kenya-based PI:

- Overall responsibility for study design and implementation
- Analysis and synthesis of data
- Liaising with local IRB, MOH and other partners in the field
- Overall responsibility for dissemination of findings to local stakeholders

Paul Drain, co-investigator

- Contribute to study design and implementation
- Participate in data analysis
- Oversee DBS testing procedures

Other co-investigators

- Contribute to study design and implementation
- Participate in data analysis

1. **References**

   . JUNPoHA. U. The Gap Report; Adolescent Girls and Young Women. 2014. [↑](#endnote-ref-1)
2. . Kharsany ABMK, Quarraisha A. HIV Infection and AIDS in Sub-Saharan Africa: Current Status, Challenges and Opportunities. The open AIDS journal. 2016 (10):34-48. PMCID: PMC4893541. [↑](#endnote-ref-2)
3. . Abdool Karim QK, Ayesha B M ; Leask, Kerry ; Ntombela, Fanelisibonge ; Humphries, Hilton ; Frohlich, Janet A ; Samsunder, Natasha ; Grobler, Anneke ; Dellar, Rachael ; Abdool Karim, Salim S. Prevalence of HIV, HSV-2 and pregnancy among high school students in rural KwaZulu-Natal, South Africa: a bio- behavioural cross-sectional survey. Sexually Transmitted Infections. 2014;90(8):620-6. PMCID: PMC4759648. [↑](#endnote-ref-3)
4. . PEPFAR. Dreams: Working Together for an AIDS-free Future for Girls and Women. [↑](#endnote-ref-4)
5. . Grant RMA, Peter L ; Mcmahan, Vanessa ; Liu, Albert ; Amico, K Rivet ; Mehrotra, Megha ; Hosek, Sybil ; Mosquera, Carlos ; Casapia, Martin ; Montoya, Orlando ; Buchbinder, Susan ; Veloso, Valdilea G ; Mayer, Kenneth ; Chariyalertsak, Suwat ; Bekker, Linda-Gail ; Kallas, Esper G ; Schechter, Mauro ; Guanira, Juan ; Bushman, Lane ; Burns, David N ; Rooney, James F ; Glidden, David V. Uptake of pre-exposure prophylaxis, sexual practices, and HIV incidence in men and transgender women who have sex with men: a cohort study. The Lancet Infectious Diseases. 2014;14(9):820-9. PMCID: PMID 25065857. [↑](#endnote-ref-5)
6. . Grant RML, Javier R ; Anderson, Peter L ; Mcmahan, Vanessa ; Liu, Albert Y ; Vargas, Lorena ; Goicochea, Pedro ; Casapía, Martín ; Guanira - Carranza, Juan Vicente ; Ramirez - Cardich, Maria E ; Montoya - Herrera, Orlando ; Fernández, Telmo ; Veloso, Valdilea G ; Buchbinder, Susan P ; Chariyalertsak, Suwat ; Schechter, Mauro ; Bekker, Linda - Gail ; Mayer, Kenneth H ; Kallás, Esper Georges ; Amico, K. Rivet ; Mulligan, Kathleen ; Bushman, Lane R ; Hance, Robert J ; Ganoza, Carmela ; Defechereux, Patricia ; Postle, Brian ; Wang, Furong ; Mcconnell, J. Jeff ; Zheng, Jia - Hua ; Lee, Jeanny ; Rooney, James F ; Jaffe, Howard S ; Martinez, Ana I ; Burns, David N ; Glidden, David V. Preexposure Chemoprophylaxis for HIV Prevention in Men Who Have Sex with Men. The New England Journal of Medicine. 2010; 363(27):2587-99. PMCID: PMCID: PMC3079639. [↑](#endnote-ref-6)
7. . Mccormack SD, David T ; Desai, Monica ; Dolling, David I ; Gafos, Mitzy ; Gilson, Richard ; Sullivan, Ann K ; Clarke, Amanda ; Reeves, Iain ; Schembri, Gabriel ; Mackie, Nicola ; Bowman, Christine ; Lacey, Charles J ; Apea, Vanessa ; Brady, Michael ; Fox, Julie ; Taylor, Stephen ; Antonucci, Simone ; Khoo, Saye H ; Rooney, James ; Nardone, Anthony ; Fisher, Martin ; Mcowan, Alan ; Phillips, Andrew N ; Johnson, Anne M ; Gazzard, Brian ; Gill, Owen N. Pre-exposure prophylaxis to prevent the acquisition of HIV-1 infection (PROUD): effectiveness results from the pilot phase of a pragmatic open-label randomised trial. The Lancet. 2016;387(10013):53-60. PMCID: PMC4700047. [↑](#endnote-ref-7)
8. . Thigpen MCK, Poloko M ; Paxton, Lynn A ; Smith, Dawn K ; Rose, Charles E ; Segolodi, Tebogo M ; Henderson, Faith L ; Pathak, Sonal R ; Soud, Fatma A ; Chillag, Kata L ; Mutanhaurwa, Rodreck ; Chirwa, Lovemore Ian ; Kasonde, Michael ; Abebe, Daniel ; Buliva, Evans ; Gvetadze, Roman J ; Johnson, Sandra; Sukalac, Thom ; Thomas, Vasavi T ; Hart, Clyde ; Johnson, Jeffrey A ; Malotte, C. Kevin ; Hendrix, Craig W ; Brooks, John T. Antiretroviral Preexposure Prophylaxis for Heterosexual HIV Transmission in Botswana. The New England Journal of Medicine. 2012;367(5):423-34. PMCID: PMID 22784038. [↑](#endnote-ref-8)
9. . Baeten JMD, Deborah ; Ndase, Patrick ; Mugo, Nelly R ; Campbell, James D ; Wangisi, Jonathan ; Tappero, Jordan W ; Bukusi, Elizabeth A ; Cohen, Craig R ; Katabira, Elly ; Ronald, Allan ; Tumwesigye, Elioda ; Were, Edwin ; Fife, Kenneth H ; Kiarie, James ; Farquhar, Carey ; John - Stewart, Grace ; Kakia, Aloysious ; Odoyo, Josephine ; Mucunguzi, Akasiima ; Nakku - Joloba, Edith ; Twesigye, Rogers ; Ngure, Kenneth ; Apaka, Cosmas ; Tamooh, Harrison ; Gabona, Fridah ; Mujugira, Andrew ; Panteleeff, Dana ; Thomas, Katherine K ; Kidoguchi, Lara ; Krows, Meighan ; Revall, Jennifer ; Morrison, Susan ; Haugen, Harald ; Emmanuel - Ogier, Mira ; Ondrejcek, Lisa ; Coombs, Robert W ; Frenkel, Lisa ; Hendrix, Craig ; Bumpus, Namandjé N ; Bangsberg, David ; Haberer, Jessica E ; Stevens, Wendy S ; Lingappa, Jairam R ; Celum, Connie. Antiretroviral Prophylaxis for HIV Prevention in Heterosexual Men and Women. The New England Journal of Medicine. 2012;367(5):399-410. PMCID: PMC3770474. [↑](#endnote-ref-9)
10. . Marrazzo JMR, Gita ; Richardson, Barbra A ; Gomez, Kailazarid ; Mgodi, Nyaradzo ; Nair, Gonasagrie ; Palanee, Thesla ; Nakabiito, Clemensia ; Van Der Straten, Ariane ; Noguchi, Lisa ; Hendrix, Craig W ; Dai,James Y ; Ganesh, Shayhana ; Mkhize, Baningi ; Taljaard, Marthinette ; Parikh, Urvi M ; Piper, Jeanna ; Mâsse, Benoît ; Grossman, Cynthia ; Rooney, James ; Schwartz, Jill L ; Watts, Heather ; Marzinke, Mark A ; Hillier, Sharon L ; Mcgowan, Ian M ; Chirenje, Z. Mike. Tenofovir-Based Preexposure Prophylaxis for HIV Infection among African Women. The New England Journal of Medicine. 2015;372(6):509-18. PMCID: PMCID: PMC4341965. [↑](#endnote-ref-10)
11. . Anderson PLG, David V ; Liu, Albert ; Buchbinder, Susan ; Lama, Javier R ; Guanira, Juan Vicente ; Mcmahan, Vanessa ; Bushman, Lane R ; Casapía, Martín ; Montoya-Herrera, Orlando ; Veloso, Valdilea G; Mayer, Kenneth H ; Chariyalertsak, Suwat ; Schechter, Mauro ; Bekker, Linda-Gail ; Kallás, Esper Georges ; Grant, Robert M. Emtricitabine-tenofovir concentrations and pre-exposure prophylaxis efficacy in men who have sex with men. Science translational medicine. 2012;4(151):151ra25. PMCID: PMC3721979. [↑](#endnote-ref-11)
12. . Van Damme LC, Amy ; Ahmed, Khatija ; Agot, Kawango ; Lombaard, Johan ; Kapiga, Saidi ; Malahleha, Mookho ; Owino, Fredrick ; Manongi, Rachel ; Onyango, Jacob ; Temu, Lucky ; Monedi, Modie Constance ; Mak'oketch, Paul ; Makanda, Mankalimeng ; Reblin, Ilse ; Makatu, Shumani Elsie ; Saylor, Lisa ; Kiernan, Haddie ; Kirkendale, Stella ; Wong, Christina ; Grant, Robert ; Kashuba, Angela ; Nanda, Kavita ; Mandala, Justin ; Fransen, Katrien ; Deese, Jennifer ; Crucitti, Tania ; Mastro, Timothy D ; Taylor, Douglas. Preexposure Prophylaxis for HIV Infection among African Women. The New England Journal of Medicine. 2012;367(5):411-22. PMCID: PMC3687217. [↑](#endnote-ref-12)
13. . Grant RM, Lama JR, Anderson PL, McMahan V, Liu AY, Vargas L, Goicochea P, Casapia M, Guanira-Carranza JV, Ramirez-Cardich ME, Montoya-Herrera O, Fernandez T, Veloso VG, Buchbinder SP, Chariyalertsak S, Schechter M, Bekker LG, Mayer KH, Kallas EG, Amico KR, Mulligan K, Bushman LR, Hance RJ, Ganoza C, Defechereux P, Postle B, Wang F, McConnell JJ, Zheng JH, Lee J, Rooney JF, Jaffe HS, Martinez AI, Burns DN, Glidden DV and iPrEx Study Team. Preexposure chemoprophylaxis for HIV prevention in men who have sex with men. N Engl J Med. 2010;363(27):2587-99. PMCID: 3079639. [↑](#endnote-ref-13)
14. . Baeten JM, Donnell D, Ndase P, Mugo NR, Campbell JD, Wangisi J, Tappero JW, Bukusi EA, Cohen CR, Katabira E, Ronald A, Tumwesigye E, Were E, Fife KH, Kiarie J, Farquhar C, John-Stewart G, Kakia A, Odoyo J, Mucunguzi A, Nakku-Joloba E, Twesigye R, Ngure K, Apaka C, Tamooh H, Gabona F, Mujugira A, Panteleeff D, Thomas KK, Kidoguchi L, Krows M, Revall J, Morrison S, Haugen H, Emmanuel-Ogier M, Ondrejcek L, Coombs RW, Frenkel L, Hendrix C, Bumpus NN, Bangsberg D, Haberer JE, Stevens WS, Lingappa JR, Celum C and Partners Pr EPST. Antiretroviral prophylaxis for HIV prevention in heterosexual men and women. N Engl J Med. 2012;367(5):399-410. PMCID: 3770474. [↑](#endnote-ref-14)
15. . Thigpen MC, Kebaabetswe PM, Paxton LA, Smith DK, Rose CE, Segolodi TM, Henderson FL, Pathak SR, Soud FA, Chillag KL, Mutanhaurwa R, Chirwa LI, Kasonde M, Abebe D, Buliva E, Gvetadze RJ, Johnson S, Sukalac T, Thomas VT, Hart C, Johnson JA, Malotte CK, Hendrix CW, Brooks JT and TDF Study Group. Antiretroviral preexposure prophylaxis for heterosexual HIV transmission in Botswana. N Engl J Med. 2012;367(5):423-34. [↑](#endnote-ref-15)
16. . Choopanya K, Martin M, Suntharasamai P, Sangkum U, Mock PA, Leethochawalit M, Chiamwongpaet S, Kitisin P, Natrujirote P, Kittimunkong S, Chuachoowong R, Gvetadze RJ, McNicholl JM, Paxton LA, Curlin ME, Hendrix CW, Vanichseni S and Bangkok Tenofovir Study Group. Antiretroviral prophylaxis for HIV infection in injecting drug users in Bangkok, Thailand (the Bangkok Tenofovir Study): a randomised, double-blind, placebo-controlled phase 3 trial. Lancet. 2013;381(9883):2083-90. [↑](#endnote-ref-16)
17. . Haberer JE, Baeten JM, Campbell J, Wangisi J, Katabira E, Ronald A, Tumwesigye E, Psaros C, Safren SA, Ware NC, Thomas KK, Donnell D, Krows M, Kidoguchi L, Celum C and Bangsberg DR. Adherence to antiretroviral prophylaxis for HIV prevention: a substudy cohort within a clinical trial of serodiscordant couples in East Africa. PLoS Med. 2013;10(9):e1001511. [↑](#endnote-ref-17)
18. . Marrazzo JM, Ramjee G, Richardson BA, Gomez K, Mgodi N, Nair G, Palanee T, Nakabiito C, van der Straten A, Noguchi L, Hendrix CW, Dai JY, Ganesh S, Mkhize B, Taljaard M, Parikh UM, Piper J, Masse B, Grossman C, Rooney J, Schwartz JL, Watts H, Marzinke MA, Hillier SL, McGowan IM, Chirenje ZM and Voice Study Team. Tenofovir-based preexposure prophylaxis for HIV infection among African women. N Engl J Med. 2015;372(6):509-18. PMCID: 4341965. [↑](#endnote-ref-18)
19. . Van Damme L, Corneli A, Ahmed K, Agot K, Lombaard J, Kapiga S, Malahleha M, Owino F, Manongi R, Onyango J, Temu L, Monedi MC, Mak'Oketch P, Makanda M, Reblin I, Makatu SE, Saylor L, Kiernan H, Kirkendale S, Wong C, Grant R, Kashuba A, Nanda K, Mandala J, Fransen K, Deese J, Crucitti T, Mastro TD, Taylor D and FEM-PrEP Study Group. Preexposure prophylaxis for HIV infection among African women. N Engl J Med. 2012;367(5):411-22. PMCID: 3687217. [↑](#endnote-ref-19)
20. . Chan PA, Mena L, Patel R, Oldenburg CE, Beauchamps L, Perez-Brumer AG, Parker S, Mayer KH, Mimiaga MJ and Nunn A. Retention in care outcomes for HIV pre-exposure prophylaxis implementation programmes among men who have sex with men in three US cities. J Int AIDS Soc. 2016;19(1):20903. [↑](#endnote-ref-20)
21. . Liu AY, Cohen SE, Vittinghoff E, Anderson PL, Doblecki-Lewis S, Bacon O, Chege W, Postle BS, Matheson T, Amico KR, Liegler T, Rawlings MK, Trainor N, Blue RW, Estrada Y, Coleman ME, Cardenas G, Feaster DJ, Grant R, Philip SS, Elion R, Buchbinder S and Kolber MA. Preexposure Prophylaxis for HIV Infection Integrated With Municipal- and Community-Based Sexual Health Services. JAMA Intern Med. 2016;176(1):75-84. [↑](#endnote-ref-21)
22. . Hosek SG, Siberry G, Bell M, Lally M, Kapogiannis B, Green K, Fernandez MI, Rutledge B, Martinez J, Garofalo R and Wilson CM. The acceptability and feasibility of an HIV preexposure prophylaxis (PrEP) trial with young men who have sex with men. J Acquir Immune Defic Syndr. 2013;62(4):447-56. [↑](#endnote-ref-22)
23. . Deutsch MB, Glidden DV, Sevelius J, Keatley J, McMahan V, Guanira J, Kallas EG, Chariyalertsak S and Grant RM. HIV pre-exposure prophylaxis in transgender women: a subgroup analysis of the iPrEx trial. Lancet HIV. 2015;2(12):e512-9. PMCID: 5111857. [↑](#endnote-ref-23)
24. . Fogarty L, Roter D, Larson S, Burke J, Gillespie J and Levy R. Patient adherence to HIV medication regimens: a review of published and abstract reports. Patient Educ Couns. 2002;46(2):93-108. [↑](#endnote-ref-24)
25. . Barnighausen T, Chaiyachati K, Chimbindi N, Peoples A, Haberer J and Newell ML. Interventions to increase antiretroviral adherence in sub-Saharan Africa: a systematic review of evaluation studies. Lancet Infect Dis. 2011;11(12):942-51. PMCID: 4250825. [↑](#endnote-ref-25)
26. . Kalichman SC, Amaral CM, Cherry C, Flanagan J, Pope H, Eaton L, Kalichman MO, Cain D, Detorio M, Caliendo A and Schinazi RF. Monitoring medication adherence by unannounced pill counts conducted by telephone: reliability and criterion-related validity. HIV Clin Trials. 2008;9(5):298-308. PMCID: 2937191. [↑](#endnote-ref-26)
27. . Kalichman SC, Amaral C, Swetsze C, Eaton L, Kalichman MO, Cherry C, Detorio M, Caliendo AM and Schinazi RF. Monthly unannounced pill counts for monitoring HIV treatment adherence: tests for self-monitoring and reactivity effects. HIV Clin Trials. 2010;11(6):325-31. [↑](#endnote-ref-27)
28. . Biressaw S, Abegaz WE, Abebe M, Taye WA and Belay M. Adherence to Antiretroviral Therapy and associated factors among HIV infected children in Ethiopia: unannounced home-based pill count versus caregivers' report. BMC Pediatr. 2013;13:132. PMCID: 3766076. [↑](#endnote-ref-28)
29. . Sigaloff KC, Hamers RL, Wallis CL, Kityo C, Siwale M, Ive P, Botes ME, Mandaliya K, Wellington M, Osibogun A, Stevens WS, van Vugt M, de Wit TF and PharmAccess African Studies to Evaluate Resistance. Unnecessary antiretroviral treatment switches and accumulation of HIV resistance mutations; two arguments for viral load monitoring in Africa. J Acquir Immune Defic Syndr. 2011;58(1):23-31. [↑](#endnote-ref-29)
30. . Haberer JE. Current concepts for PrEP adherence in the PrEP revolution: from clinical trials to routine practice. Curr Opin HIV AIDS. 2016;11(1):10-7. [↑](#endnote-ref-30)
31. . Grant RM, Anderson PL, McMahan V, Liu A, Amico KR, Mehrotra M, Hosek S, Mosquera C, Casapia M, Montoya O, Buchbinder S, Veloso VG, Mayer K, Chariyalertsak S, Bekker LG, Kallas EG, Schechter M, Guanira J, Bushman L, Burns DN, Rooney JF, Glidden DV and IPrEx Study Team. Uptake of pre-exposure prophylaxis, sexual practices, and HIV incidence in men and transgender women who have sex with men: a cohort study. Lancet Infect Dis. 2014;14(9):820-9. [↑](#endnote-ref-31)
32. . Gandhi M, Glidden DV, Liu A, Anderson PL, Horng H, Defechereux P, Guanira JV, Grinsztejn B, Chariyalertsak S, Bekker LG and Grant RM. Strong Correlation Between Concentrations of Tenofovir (TFV) Emtricitabine (FTC) in Hair and TFV Diphosphate and FTC Triphosphate in Dried Blood Spots in the iPrEx Open Label Extension: Implications for Pre-exposure Prophylaxis Adherence Monitoring. J Infect Dis. 2015;212(9):1402-6. PMCID: 4601920. [↑](#endnote-ref-32)
33. . Liu H, Golin CE, Miller LG, Hays RD, Beck CK, Sanandaji S, Christian J, Maldonado T, Duran D, Kaplan AH and Wenger NS. A comparison study of multiple measures of adherence to HIV protease inhibitors. Ann Intern Med. 2001;134(10):968-77. [↑](#endnote-ref-33)
34. . Castillo-Mancilla JR, Zheng JH, Rower JE, Meditz A, Gardner EM, Predhomme J, Fernandez C, Langness J, Kiser JJ, Bushman LR and Anderson PL. Tenofovir, emtricitabine, and tenofovir diphosphate in dried blood spots for determining recent and cumulative drug exposure. AIDS Res Hum Retroviruses. 2013;29(2):384-90. PMCID: 3552442. [↑](#endnote-ref-34)
35. . Gandhi M, Glidden DV, Liu A, Anderson PL, Horng H, Defechereux P, Guanira JV, Grinsztejn B, Chariyalertsak S, Bekker LG, Grant RM. Strong Correlation Between Concentrations of Tenofovir (TFV) Emtricitabine (FTC) in Hair and TFV Diphosphate and FTC Triphosphate in Dried Blood Spots in the iPrEx Open Label Extension: Implications for Pre-exposure Prophylaxis Adherence Monitoring. J Infect Dis. 2015 Nov 1;212(9):1402-6. PubMed PMID: 25895984. PMCID: PMC4601920. Epub 2015/04/22. eng. [↑](#endnote-ref-35)
36. . Bangsberg DR. Less than 95% adherence to nonnucleoside reverse-transcriptase inhibitor therapy can lead to viral suppression. Clin Infect Dis. 2006;43(7):939-41. [↑](#endnote-ref-36)
37. . Corneli A, Perry B, Agot K, Ahmed K, Malamatsho F, Van Damme L. Facilitators of adherence to the study pill in the FEM-PrEP clinical trial. PLoS One. 2015;10(4):e0125458. PubMed PMID: 25867624. PMCID: PMC4395082. Epub 2015/04/14. eng. [↑](#endnote-ref-37)
38. . Koester KA, Liu A, Eden C, Amico KR, McMahan V, Goicochea P, Hosek S, Mayer KH, Grant RM. Acceptability of drug detection monitoring among participants in an open-label pre-exposure prophylaxis study. AIDS Care. 2015;27(10):1199-204. PubMed PMID: 26001026. Epub 2015/05/23. eng. [↑](#endnote-ref-38)
39. . National Institute of Allergy and Infectious Diseases (NIAID). In Adolescents, Oral Truvada And Vaginal Ring For HIV Prevention Are Safe, Acceptable; HIV/AIDS News page. US Department of Health and Human Services AIDSinfo website.https://www.niaid.nih.gov/news-events/adolescents-oral-truvada-and-vaginal-ring-hiv-prevention-are-safe-acceptable/; July 25, 2017. [↑](#endnote-ref-39)
40. . Auerbach, JD and Hoppe, TA. Beyond ‘‘getting drugs into bodies’’: social science perspectives on pre-exposure prophylaxis for HIV. Journal of the International AIDS Society 2015, 18(Suppl 3):19983 [↑](#endnote-ref-40)
41. . Venter WDF; Cowan F; Black V; Rebe L; Bekker, LG. Pre-exposure prophylaxis in Southern Africa: feasible or not? Journal of the International AIDS Society 2015, 18(Suppl 3):19979 [↑](#endnote-ref-41)
42. . Celum CL; Delany-Moretlwe S; McConnell M; van Rooyen H; Bekker LG; Kurth A; Bukusi E; Desmond C; Morton J; Baeten JM. Rethinking HIV prevention to prepare for oral PrEP implementation for young African women. Journal of the International AIDS Society 2015, 18(Suppl 3):20227 [↑](#endnote-ref-42)
43. . Amico KR, Mehrotra M, Avelino-Silva VI, McMahan V, Veloso VG, Anderson P, Guanira J, Grant R. Self-reported Recent PrEP Dosing and Drug Detection in an Open Label PrEP Study. AIDS Behav. 2016 Jul;20(7):1535-40. PubMed PMID: 26992393. PMCID: PMC4903107. Epub 2016/03/20. eng. [↑](#endnote-ref-43)
44. . Corneli AL, McKenna K, Perry B, Ahmed K, Agot K, Malamatsho F, Skhosana J, Odhiambo J, Van Damme L. The science of being a study participant: FEM-PrEP participants' explanations for overreporting adherence to the study pills and for the whereabouts of unused pills. J Acquir Immune Defic Syndr. 2015 Apr 15;68(5):578-84. PubMed PMID: 25761233. Epub 2015/03/12. eng. [↑](#endnote-ref-44)
45. . Cressey TR, Avihingsanon A, Halue G, Leenasirimakul P, Sukrakanchana PO, Tawon Y, Jaisieng N, Jourdain G, Podany AT, Fletcher CV, Klinbuayaem V, Bowonwatanuwong C. Plasma and Intracellular Pharmacokinetics of Tenofovir Disoproxil Fumarate 300 mg Every 48 Hours vs 150 mg Once Daily in HIV-Infected Adults With Moderate Renal Function Impairment. Clinical infectious diseases : an official publication of the Infectious Diseases Society of America. 2015 Aug 15;61(4):633-9. PubMed PMID: 25921689. PMCID: PMC4607735. Epub 2015/04/30. eng. [↑](#endnote-ref-45)
46. . UNAIDS. HIV prevention among adolescent girls and young women. Putting HIV prevention among adolescent girls and young women on the Fast-Track and engaging men and boys [Guide]. 2016. [↑](#endnote-ref-46)
47. . Padgett, Deborah K: 2012. Qualitative and Mixed Methods in Public Health. Sage Publications, Inc. [↑](#endnote-ref-47)
48. . Watson PF, Petrie A. Method agreement analysis: a review of correct methodology. Theriogenology. 2010; 73: 1167-1179. [↑](#endnote-ref-48)
49. Kwiecien R, Kopp-Schneider A, Blettner M. Concordance analysis- part 16 of a series on evaluation of scientific publications. Dtsch Arztebl Int 2011; 108(30):515-521. [↑](#endnote-ref-49)
