# Supplementary material for: Continued attendance in a PrEP program despite low adherence and non-protective drug levels among adolescent girls and young women in Kenya: Results from a prospective cohort study
Source: PLoS Med. 2022 Sep 12;19(9):e1004097. doi: 10.1371/journal.pmed.1004097 (PMC9521917; doi:10.1371/journal.pmed.1004097)
Supplement: S1 Table — (DOCX) [file pmed.1004097.s003.docx]

**S1 Table.** Number of participants by TFV-DP levels at Interviews 1 and 2.

**Table A.** Number of participants by TFV-DP levels at Interviews 1 and 2 (All participants)

| **TFV-DP level** | **All participants** | |
| --- | --- | --- |
|  | **Provided DBS at Interview 1** | **Persisted** |
| <10 fmol/punch | 258 | 144 |
| 10-199 fmol/punch | 24 | 10 |
| 200-349 fmol/punch | 18 | 9 |
| 350-699 fmol/punch | 15 | 6 |
| 700+ fmol/punch | 21 | 7 |

TFV-DP: tenofovir-diphosphate.

**Table B.** Number of participants by TFV-DP levels at Interviews 1 and 2 (Participants with TFV-DP≥ 10fmol/punch at Interview 1).

| **TFV-DP level** | **TFV-DP≥ 10fmol/punch at Interview 1** | |
| --- | --- | --- |
|  | **Provided DBS at Interview 1** | **Persisted** |
| <10 fmol/punch | - | 29 |
| 10-199 fmol/punch | 24 | 5 |
| 200-349 fmol/punch | 18 | 8 |
| 350-699 fmol/punch | 15 | 5 |
| 700+ fmol/punch | 21 | 5 |

TFV-DP: tenofovir-diphosphate.
