# Supplementary material for: Continued attendance in a PrEP program despite low adherence and non-protective drug levels among adolescent girls and young women in Kenya: Results from a prospective cohort study
Source: PLoS Med. 2022 Sep 12;19(9):e1004097. doi: 10.1371/journal.pmed.1004097 (PMC9521917; doi:10.1371/journal.pmed.1004097)
Supplement: S2 Table — (DOCX) [file pmed.1004097.s004.docx]

**S2 Table.** Reason for stopping PrEP among AGYW reporting PrEP discontinuation and experiences taking PrEP among AGYW reporting PrEP continuation at the second interview.

| **Reason for stopping PrEP among AGYW reporting PrEP discontinuation at Interview 2 (N=105)*** | | |
| --- | --- | --- |
| **Reason** | **N** | **col %** |
| Stock out of PrEP | 26 | 24.8 |
| No longer at risk of HIV | 19 | 18.1 |
| Returned to school/was taking exam | 17 | 16.2 |
| Side effects | 16 | 15.2 |
| Sick/ caring for sick | 13 | 12.4 |
| Travelled/away from home | 12 | 11.4 |
| Pill packaging, characteristics | 6 | 5.7 |
| Daily pill taking is hard | 4 | 3.8 |
| Pregnant/breastfeeding | 4 | 3.8 |
| Pressure from partner | 4 | 3.8 |
| Health effects | 4 | 3.8 |
| Lifestyle demands | 3 | 2.9 |
| **Experiences taking PrEP among AGYW reporting PrEP continuation at Interview 2 (N=197)*** | | |
| **Things that made taking PrEP difficult** | **N** | **col %** |
| No difficulty | 106 | 53.8 |
| Forgot | 29 | 14.7 |
| Was away from home | 21 | 10.7 |
| Worried about/had side effects | 15 | 7.6 |
| Ran out | 15 | 7.6 |
| Did not have pills when needed | 9 | 4.6 |
| Fell sick | 8 | 4.1 |
| **Things that made taking PrEP easy** | **N** | **col %** |
| Committed to preventing HIV | 179 | 90.9 |
| Fear of getting HIV | 31 | 15.7 |
| Kept pills available | 31 | 15.7 |
| Used reminder strategy | 27 | 13.7 |
| Worked pill-taking into something doing anyway | 15 | 7.6 |

*Numbers and percentages do not sum to 100% as participants could choose multiple answers.

PrEP: pre-exposure prophylaxis.

AGYW: adolescent girls and young women
