# Supplementary material for: Continued attendance in a PrEP program despite low adherence and non-protective drug levels among adolescent girls and young women in Kenya: Results from a prospective cohort study
Source: PLoS Med. 2022 Sep 12;19(9):e1004097. doi: 10.1371/journal.pmed.1004097 (PMC9521917; doi:10.1371/journal.pmed.1004097)
Supplement: S3 Table — (DOCX) [file pmed.1004097.s005.docx]

**S3 Table.** Self-perceived risk for HIV infection among PrEP continuers versus discontinuers

| **Reported chance of getting HIV if not taking PrEP** | | **Reported chance of getting HIV currently** | | | | | | | | |
| --- | --- | --- | --- | --- | --- | --- | --- | --- | --- | --- |
|  |  | **No chance** | | **Small chance** | | **Moderate chance** | | **High chance** | | **Total** |
|  |  | **N** | **row**  **%** | **N** | **row %** | **N** | **row %** | **N** | **row %** | **N** |
| **Continuers** | **No chance** | 3 | 100 | 0 | 0 | 0 | 0 | 0 | 0 | 3 |
|  | **Small chance** | 20 | 74.1 | 7 | 25.9 | 0 | 0 | 0 | 0 | 27 |
|  | **Moderate chance** | 22 | 61.1 | 14 | 38.9 | 0 | 0 | 0 | 0 | 36 |
|  | **High chance** | 76 | 58.0 | 44 | 33.6 | 5 | 3.8 | 6 | 4.6 | 131 |
| **Discontinuers** | **N/A** | 30 | 28.6 | 37 | 35.2 | 15 | 14.3 | 23 | 21.9 | 105 |
| **Total** | | 151 | 50.0 | 102 | 33.8 | 20 | 6.6 | 29 | 9.6 | 302 |

PrEP: pre-exposure prophylaxis.
