# Supplementary material for: Continued attendance in a PrEP program despite low adherence and non-protective drug levels among adolescent girls and young women in Kenya: Results from a prospective cohort study
Source: PLoS Med. 2022 Sep 12;19(9):e1004097. doi: 10.1371/journal.pmed.1004097 (PMC9521917; doi:10.1371/journal.pmed.1004097)
Supplement: S4 Table — (DOCX) [file pmed.1004097.s006.docx]

**S4 Table.** Characteristics of persisters with TFV-DP <10 vs. ≥10 fmol/punch at the second interview

|  | **Persisters with TFV-DP <10 fmol***  **(N=144)** | **Persisters with TFV-DP ≥10 fmol**  **(N=32)** | **Univariable analysis^1^** | | **Multivariable analysis^2^** | |
| --- | --- | --- | --- | --- | --- | --- |
| **Characteristic** | **n** | **n** | **Odds ratio**  **OR**  **[95%CI]** | **p-value** | **Odds ratio OR**  **[95%CI]** | **p-value** |
| Age ≥22 years | 100 | 19 | 0.63 [ 0.27 , 1.47 ] | 0.286 | 0.66 [ 0.29 , 1.46 ] | 0.304 |
| Still active in the DREAMS program | 132 | 28 | 0.83 [ 0.38 , 1.82 ] | 0.639 | 0.74 [ 0.38 , 1.46 ] | 0.389 |
| Currently has a sexual partner | 140 | 31 | 0.83 [ 0.1 , 6.54 ] | 0.858 | 1 [ 0.13 , 7.92 ] | 0.998 |
| Currently has multiple sex partners | 23 | 5 | 1.07 [ 0.48 , 2.38 ] | 0.860 | 0.95 [ 0.48 , 1.91 ] | 0.895 |
| Married/cohabiting | 67 | 15 | 0.7 [ 0.39 , 1.27 ] | 0.242 | 1.05 [ 0.48 , 2.31 ] | 0.908 |
| One or more children | 110 | 22 | 0.55 [ 0.28 , 1.1 ] | 0.090 | 0.68 [ 0.33 , 1.4 ] | 0.293 |
| Lives with parents or grandparents | 68 | 11 | 1.16 [ 0.69 , 1.96 ] | 0.575 | 0.66 [ 0.34 , 1.31 ] | 0.237 |
| Lives with partner | 66 | 15 | 0.71 [ 0.38 , 1.33 ] | 0.286 | 1.06 [ 0.47 , 2.39 ] | 0.882 |
| Partner is aware of PrEP use | 88 | 20 | 0.94 [ 0.5 , 1.76 ] | 0.846 | 0.99 [ 0.49 , 2.01 ] | 0.982 |
| Partner is very supportive of PrEP use | 58 | 13 | 1.07 [ 0.51 , 2.26 ] | 0.859 | 1.02 [ 0.48 , 2.17 ] | 0.957 |
| Partner is HIV positive | 6 | 4 | 3.02 [ 0.9 , 10.18 ] | 0.075 | 3.14 [ 0.94 , 10.42 ] | 0.062 |
| Partner has other partners | 41 | 9 | 1.03 [ 0.58 , 1.82 ] | 0.924 | 1.1 [ 0.6 , 2.01 ] | 0.762 |
| AGYW believes partner puts her at risk | 67 | 13 | 0.79 [ 0.48 , 1.3 ] | 0.354 | 0.91 [ 0.52 , 1.59 ] | 0.730 |
| Moderate-to-high HIV chance if not taking PrEP | 123 | 28 | 1.26 [ 0.42 , 3.79 ] | 0.678 | 1.35 [ 0.47 , 3.87 ] | 0.576 |
| Experience of intimate partner violence (IPV score >10) | 10 | 0 | 0 | . |  | . |
| Depression, moderate to severe | 10 | 5 | 2.89 [ 1.36 , 6.13 ] | 0.006 | 2.22 [ 0.96 , 5.15 ] | 0.064 |
| Social support (most or all the time) | 27 | 2 | 0.38 [ 0.13 , 1.14 ] | 0.085 | 0.31 [ 0.09 , 1.03 ] | 0.055 |
| Inconsistent or no condom use | 116 | 23 | 0.55 [ 0.27 , 1.11 ] | 0.093 | 0.66 [ 0.32 , 1.35 ] | 0.257 |
| Contraceptive use, any | 109 | 26 | 1.14 [ 0.48 , 2.7 ] | 0.773 | 1.1 [ 0.48 , 2.53 ] | 0.817 |
| oral | 7 | 2 | 1.14 [ 0.26 , 4.94 ] | 0.859 | 1.19 [ 0.23 , 6.27 ] | 0.838 |
| injectable | 30 | 9 | 0.87 [ 0.47 , 1.6 ] | 0.657 | 1.27 [ 0.67 , 2.41 ] | 0.472 |
| Implant | 44 | 7 | 0.68 [ 0.3 , 1.56 ] | 0.367 | 0.63 [ 0.28 , 1.41 ] | 0.261 |
| male condoms | 26 | 8 | 1.99 [ 1.15 , 3.46 ] | 0.014 | 1.5 [ 0.83 , 2.72 ] | 0.178 |
| female condoms | 2 | 0 |  | . |  | . |
| Friends are on PrEP | 138 | 28 | 0.4 [ 0.08 , 1.86 ] | 0.241 | 0.33 [ 0.07 , 1.54 ] | 0.158 |
| Told someone of PrEP use since Interview 1 | 76 | 19 | 1.41 [ 0.62 , 3.23 ] | 0.412 | 1.37 [ 0.57 , 3.3 ] | 0.487 |
| Months since PrEP initiation at interview1, 2-3 months | 37 | 8 | 1.59 [ 1.12 , 2.26 ] | 0.009 | 1.11 [ 0.82 , 1.51 ] | 0.504 |
| Months since PrEP initiation at Interview 1, 4-6 months | 76 | 20 | 0.99 [ 0.46 , 2.12 ] | 0.980 | 1.27 [ 0.65 , 2.47 ] | 0.484 |
| Months since PrEP initiation at Interview1, 6 + months | 31 | 4 | 0.33 [ 0.15 , 0.73 ] | 0.006 | 0.51 [ 0.21 , 1.24 ] | 0.136 |
| Education, primary school | 46 | 15 | 1.62 [ 0.82 , 3.19 ] | 0.165 | 1.86 [ 0.98 , 3.54 ] | 0.059 |
| Education, secondary school | 85 | 11 | 0.53 [ 0.26 , 1.09 ] | 0.083 | 0.42 [ 0.22 , 0.81 ] | 0.010 |
| Education, postsecondary | 13 | 6 | 1.8 [ 0.84 , 3.88 ] | 0.132 | 1.64 [ 0.71 , 3.78 ] | 0.247 |
| Currently in school | 44 | 11 | 1.2 [ 0.63 , 2.31 ] | 0.577 | 1.19 [ 0.58 , 2.48 ] | 0.633 |
| In PrEP support group | 118 | 24 | 0.66 [ 0.25 , 1.79 ] | 0.416 | 0.68 [ 0.26 , 1.76 ] | 0.425 |

* TFV-DP level consistent with no PrEP use in the recent past

^1^Odds ratio and corresponding p-value were based on univariable generalized estimating equations with logit link function in the model accounting for clustering of study participants within wards.

^2^Odds ratio and corresponding p-value were based on multivariable generalized estimating equations with logit link function in the model adjusted for county of residence as well as clustering of study participants within wards.

TFV-DP: tenofovir-diphosphate. PrEP: pre-exposure prophylaxis.

AGYW: adolescent girls and young women.
