# Supplementary material for: Continued attendance in a PrEP program despite low adherence and non-protective drug levels among adolescent girls and young women in Kenya: Results from a prospective cohort study
Source: PLoS Med. 2022 Sep 12;19(9):e1004097. doi: 10.1371/journal.pmed.1004097 (PMC9521917; doi:10.1371/journal.pmed.1004097)
Supplement: S5 Table — (DOCX) [file pmed.1004097.s007.docx]

**S5 Table.** Factors associated with Persistence (Discontinuers versus Persisters)

|  | **Discontinuers**  **(N=105)** | **Persisters**  **(N=176)** | **Univariable analysis^1^** | | **Multivariable analysis^2^** | |
| --- | --- | --- | --- | --- | --- | --- |
| **Factor** | **n** | **n** | **Odds ratio OR[95%CI]** | **p-value** | **Odds ratio**  **OR [95%CI]** | **p-value** |
| **Age ≥22 years** | 56 | 119 | 1.78 [ 1.04 , 3.04 ] | 0.035 | 2.08 [ 1.09 , 3.98 ] | 0.027 |
| Still active in the DREAMS program | 77 | 160 | 3.85 [ 1.57 , 9.41 ] | 0.003 | 3.03 [ 0.96 , 9.6 ] | 0.059 |
| Currently has a sexual partner | 100 | 171 | 1.68 [ 0.55 , 5.12 ] | 0.361 |  | . |
| Currently has multiple sex partners | 7 | 28 | 2.96 [ 1.21 , 7.27 ] | 0.018 | 2.87 [ 0.67 , 12.28 ] | 0.155 |
| Married/cohabiting | 44 | 82 | 1.18 [ 0.71 , 1.97 ] | 0.515 |  | . |
| One or more children | 68 | 132 | 1.62 [ 0.96 , 2.72 ] | 0.069 | 0.92 [ 0.37 , 2.27 ] | 0.860 |
| Lives with parents or grandparents | 54 | 79 | 0.78 [ 0.45 , 1.35 ] | 0.376 |  | . |
| Lives with partner | 43 | 81 | 1.21 [ 0.71 , 2.06 ] | 0.489 |  | . |
| Partner is aware of PrEP use | 51 | 108 | 1.84 [ 1.1 , 3.06 ] | 0.020 | 2.04 [ 0.95 , 4.37 ] | 0.066 |
| Partner is very supportive of PrEP use | 23 | 71 | 2.52 [ 1.72 , 3.71 ] | 0.000 | 2.1 [ 0.99 , 4.49 ] | 0.054 |
| Partner is HIV positive | 1 | 10 | 6.15 [ 0.97 , 39.19 ] | 0.054 | 1.93 [ 0.37 , 9.97 ] | 0.432 |
| Partner has other partners | 16 | 50 | 2.2 [ 0.98 , 4.96 ] | 0.058 | 1.27 [ 0.56 , 2.85 ] | 0.566 |
| AGYW believes partner puts her at risk | 30 | 80 | 2.09 [ 1.07 , 4.11 ] | 0.032 | 0.68 [ 0.28 , 1.64 ] | 0.388 |
| **Moderate-to-high HIV chance if not taking PrEP** | 38 | 151 | 10.6 [ 5.78 , 19.43 ] | <0.001 | 10.69 [ 5.7 , 20.04 ] | <0.001 |
| Experience of intimate partner violence (IPV score >10) | 7 | 10 | 0.81 [ 0.31 , 2.09 ] | 0.664 |  | . |
| Depression, moderate to severe | 9 | 15 | 1.02 [ 0.5 , 2.08 ] | 0.948 |  | . |
| Social support (most or all the time) | 24 | 29 | 0.67 [ 0.41 , 1.11 ] | 0.122 |  | . |
| Inconsistent or no condom use | 82 | 139 | 1.04 [ 0.67 , 1.63 ] | 0.848 |  | . |
| Contraceptive use, any | 77 | 135 | 1.21 [ 0.72 , 2.05 ] | 0.473 |  | . |
| oral | 5 | 9 | 1.09 [ 0.3 , 3.98 ] | 0.895 |  | . |
| **injectable** | 13 | 39 | 1.99 [ 1.15 , 3.44 ] | 0.014 | 2.56 [ 1.22 , 5.35 ] | 0.012 |
| Implant | 21 | 51 | 1.64 [ 0.81 , 3.29 ] | 0.167 |  | . |
| male condoms | 35 | 34 | 0.48 [ 0.28 , 0.84 ] | 0.010 | 0.95 [ 0.36 , 2.47 ] | 0.912 |
| female condoms | 2 | 2 | 0.57 [ 0.09 , 3.55 ] | 0.544 |  | . |
| Friends are on PrEP | 94 | 166 | 1.99 [ 0.75 , 5.3 ] | 0.170 |  | . |
| Told someone of PrEP use since Interview 1 | 45 | 95 | 1.59 [ 1.06 , 2.38 ] | 0.025 | 1.14 [ 0.72 , 1.79 ] | 0.582 |
| Months since PrEP initiation at interview1, 2-3 months | 30 | 45 | 0.87 [ 0.43 , 1.75 ] | 0.693 |  | . |
| Months since PrEP initiation at Interview 1, 4-6 months | 59 | 96 | 0.92 [ 0.6 , 1.42 ] | 0.714 |  | . |
| Months since PrEP initiation at Interview1, 6 + months | 15 | 35 | 1.49 [ 0.74 , 3 ] | 0.262 |  | . |
| Education, primary school | 32 | 61 | 1.21 [ 0.72 , 2.04 ] | 0.477 |  | . |
| Education, secondary school | 57 | 96 | 1.02 [ 0.59 , 1.75 ] | 0.951 |  | . |
| Education, postsecondary | 16 | 19 | 0.66 [ 0.29 , 1.5 ] | 0.325 |  | . |
| Currently in school | 45 | 55 | 0.59 [ 0.4 , 0.89 ] | 0.011 | 0.86 [ 0.5 , 1.49 ] | 0.598 |
| In PrEP support group | 81 | 142 | 1.28 [ 0.62 , 2.64 ] | 0.496 |  | . |

^1^Odds ratio and corresponding p-value were based on univariable generalized estimating equations with logit link function in the model accounting for clustering of study participants within wards.

^2^Odds ratio and corresponding p-value were based on multivariable generalized estimating equations with logit link function in the model adjusted for county of residence, factors with p-value<0.1 in the univariable analysis as well as clustering of study participants within wards.

PrEP: pre-exposure prophylaxis.

AGYW: adolescent girls and young women
