# Supplementary material for: Continued attendance in a PrEP program despite low adherence and non-protective drug levels among adolescent girls and young women in Kenya: Results from a prospective cohort study
Source: PLoS Med. 2022 Sep 12;19(9):e1004097. doi: 10.1371/journal.pmed.1004097 (PMC9521917; doi:10.1371/journal.pmed.1004097)
Supplement: S6 Table — (DOCX) [file pmed.1004097.s008.docx]

**S6 Table** Factors associated with Persistence (Non-persisters versus Persisters)

|  | **Non-persisters**  **(N=126)** | **Persisters**  **(N=176)** | **Univariable analysis^1^** | | **Multivariable analysis^2^** | |
| --- | --- | --- | --- | --- | --- | --- |
| **Factor** | **n** | **n** | **Odds ratio OR[95%CI]** | **p-value** | **Odds ratio**  **OR[95%CI]** | **p-value** |
| **Age ≥22 years** | 62 | 119 | 1.98 [ 1.11 , 3.52 ] | 0.020 | 2.7 [ 1.43 , 5.11 ] | 0.002 |
| Still active in the DREAMS program | 96 | 160 | 3.4 [ 1.44 , 8 ] | 0.005 | 2.58 [ 0.86 , 7.72 ] | 0.091 |
| Currently has a sexual partner | 121 | 171 | 1.36 [ 0.45 , 4.1 ] | 0.589 |  | . |
| Currently has multiple sex partners | 8 | 28 | 3.3 [ 1.57 , 6.92 ] | 0.002 | 3.38 [ 0.92 , 12.42 ] | 0.066 |
| Married/cohabiting | 56 | 82 | 1.05 [ 0.63 , 1.76 ] | 0.839 |  | . |
| One or more children | 82 | 132 | 1.6 [ 1 , 2.55 ] | 0.049 | 0.81 [ 0.32 , 2.05 ] | 0.652 |
| Lives with parents or grandparents | 60 | 79 | 0.91 [ 0.52 , 1.58 ] | 0.730 |  | . |
| Lives with partner | 55 | 81 | 1.07 [ 0.63 , 1.83 ] | 0.797 |  | . |
| Partner is aware of PrEP use | 64 | 108 | 1.68 [ 1 , 2.82 ] | 0.052 | 1.93 [ 0.94 , 3.96 ] | 0.075 |
| Partner is very supportive of PrEP use | 31 | 71 | 2.15 [ 1.47 , 3.15 ] | <0.001 | 1.65 [ 0.74 , 3.66 ] | 0.221 |
| Partner is HIV positive | 1 | 10 | 7.13 [ 1.19 , 42.72 ] | 0.031 | 3 [ 0.51 , 17.72 ] | 0.225 |
| Partner has other partners | 19 | 50 | 2.23 [ 1.08 , 4.59 ] | 0.030 | 1.65 [ 0.78 , 3.5 ] | 0.193 |
| AGYW believes partner puts her at risk | 37 | 80 | 2.02 [ 1.13 , 3.61 ] | 0.017 | 0.64 [ 0.33 , 1.24 ] | 0.186 |
| **Moderate-to-high HIV chance if not taking PrEP** | 54 | 151 | 8.08 [ 4.4 , 14.84 ] | <0.001 | 8.22 [ 4.5 , 14.99 ] | <0.001 |
| Experience of intimate partner violence (IPV score >10) | 10 | 10 | 0.64 [ 0.3 , 1.4 ] | 0.268 |  | . |
| Depression, moderate to severe | 12 | 15 | 0.94 [ 0.47 , 1.87 ] | 0.851 |  | . |
| Social support (most or all the time) | 28 | 29 | 0.7 [ 0.45 , 1.08 ] | 0.111 |  | . |
| Inconsistent or no condom use | 99 | 139 | 1.01 [ 0.65 , 1.58 ] | 0.956 |  | . |
| Contraceptive use, any | 97 | 135 | 1.02 [ 0.6 , 1.71 ] | 0.952 |  | . |
| oral | 5 | 9 | 1.32 [ 0.38 , 4.63 ] | 0.660 |  | . |
| **injectable** | 18 | 39 | 1.68 [ 1.04 , 2.71 ] | 0.033 | 2.36 [ 1.15 , 4.84 ] | 0.020 |
| Implant | 30 | 51 | 1.32 [ 0.74 , 2.38 ] | 0.348 |  | . |
| male condoms | 41 | 34 | 0.51 [ 0.31 , 0.84 ] | 0.008 | 0.89 [ 0.37 , 2.14 ] | 0.796 |
| female condoms | 2 | 2 | 0.67 [ 0.11 , 3.98 ] | 0.656 |  | . |
| Friends are on PrEP | 110 | 166 | 2.45 [ 1.09 , 5.52 ] | 0.030 | 1.5 [ 0.58 , 3.86 ] | 0.402 |
| Told someone of PrEP use since Interview 1 | 53 | 95 | 1.7 [ 1.15 , 2.51 ] | 0.008 | 1.29 [ 0.78 , 2.13 ] | 0.313 |
| Months since PrEP initiation at interview1, 2-3 months | 34 | 45 | 0.94 [ 0.52 , 1.73 ] | 0.853 |  | . |
| Months since PrEP initiation at Interview 1, 4-6 months | 69 | 96 | 0.99 [ 0.67 , 1.46 ] | 0.952 |  | . |
| Months since PrEP initiation at Interview1, 6 + months | 19 | 35 | 1.37 [ 0.72 , 2.59 ] | 0.338 |  | . |
| Education, primary school | 39 | 61 | 1.18 [ 0.73 , 1.93 ] | 0.498 |  | . |
| Education, secondary school | 70 | 96 | 0.97 [ 0.57 , 1.64 ] | 0.895 |  | . |
| Education, postsecondary | 17 | 19 | 0.77 [ 0.33 , 1.8 ] | 0.541 |  | . |
| Currently in school | 53 | 55 | 0.6 [ 0.44 , 0.83 ] | 0.002 | 1 [ 0.58 , 1.72 ] | 0.994 |
| In PrEP support group | 99 | 142 | 1.21 [ 0.61 , 2.4 ] | 0.592 |  | . |

^1^Odds ratio and corresponding p-value were based on univariable generalized estimating equations with logit link function in the model accounting for clustering of study participants within wards.

^2^Odds ratio and corresponding p-value were based on multivariable generalized estimating equations with logit link function in the model adjusted for county of residence, factors with p-value<0.1 in the univariable analysis as well as clustering of study participants within wards.

PrEP: pre-exposure prophylaxis.

AGYW: adolescent girls and young women
