# Supplementary material for: Continued attendance in a PrEP program despite low adherence and non-protective drug levels among adolescent girls and young women in Kenya: Results from a prospective cohort study
Source: PLoS Med. 2022 Sep 12;19(9):e1004097. doi: 10.1371/journal.pmed.1004097 (PMC9521917; doi:10.1371/journal.pmed.1004097)
Supplement: S1 Fig — (DOCX) [file pmed.1004097.s009.docx]

**S1 Fig.** TFV-DP levels among study participants at the first interview *(*Timepoint 1) and disposition at the time of the second interview (Timepoint 2)


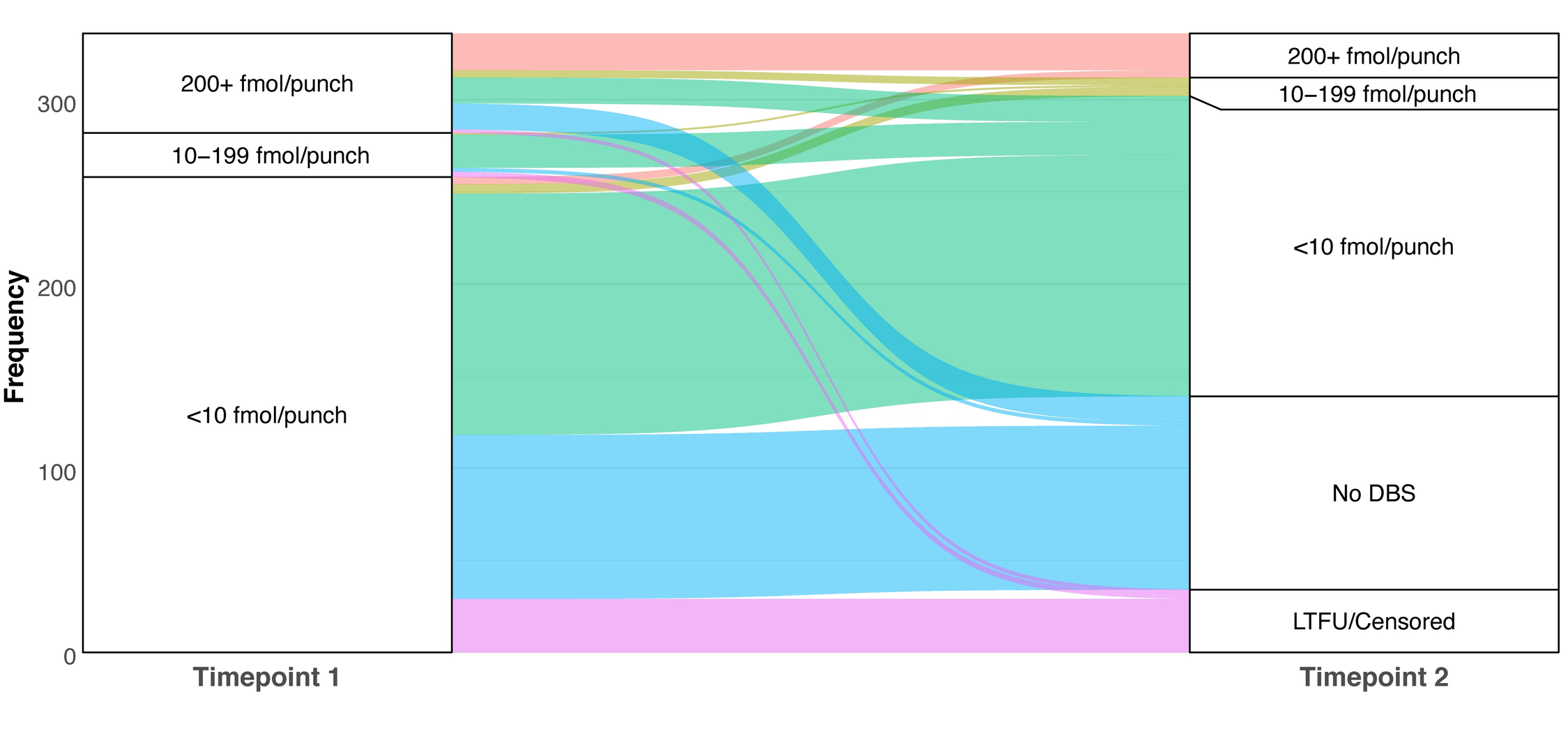


LTFU: lost-to-follow up. TFV-DP: tenofovir-diphosphate.
