# Supplementary material for: Continued attendance in a PrEP program despite low adherence and non-protective drug levels among adolescent girls and young women in Kenya: Results from a prospective cohort study
Source: PLoS Med. 2022 Sep 12;19(9):e1004097. doi: 10.1371/journal.pmed.1004097 (PMC9521917; doi:10.1371/journal.pmed.1004097)
Supplement: Attachment — Submitted filename: Response to reviewers final.docx [file pmed.1004097.s010.docx]

Requests from the editors:

1)    Please revise your title according to PLOS Medicine's style. Your title must be nondeclarative and not a question. It should begin with main concept if possible. Please place the study design in the subtitle (i.e., after a colon), e.g., a prospective cohort study.

*The title has been changed to:*

*Continued attendance in a PrEP program despite low adherence and non-protective drug levels among adolescent girls and young women in Kenya: results from a prospective cohort study*

2)    Please include line numbers in your next draft.

*Included in the text of the paper*

3)    Under the list of authors please include a footnote that indicates that the first 3 authors are joint first authors, in keeping with your prior request to make them co-/first authors.

*Included*

4)    The Data Availability Statement (DAS) requires revision. For each data source used in your study:
a) If the data are owned by a third party but freely available upon request, please note this and state the owner of the data set and contact information for data requests (web or email address). Note that a study author cannot be the contact person for the data.
b) If the data are not freely available, please describe briefly the ethical, legal, or contractual restriction that prevents you from sharing it. Please also include an appropriate contact (web or email address) for inquiries (again, this cannot be a study author).

*The dataset used for this analysis will be uploaded into Harvard Dataverse (https://dataverse.harvard.edu/ ) as soon as the paper has been published. It will contain the data collected as part of this study (**Interviews and TFV-DP levels) as well as selected variables from the DREAMS dataset pertaining to PrEP initiation and refill visits.*

5)    Abstract:
a)    Please structure your abstract using the PLOS Medicine headings (Background, Methods and Findings, Conclusions).
i)    Please combine the Methods and Findings sections into one section, “Methods and findings”.
ii)    The interpretation should be renamed “Conclusions”
b)    Please include years during which the study took place
c)    Please include the important dependent variables that are adjusted for in the analyses.
d)    Please ensure that all numbers presented in the abstract are present and identical to numbers presented in the main manuscript text.
e)    Please quantify the main results (please present both 95% CIs and p values).
f)    In the last sentence of the Abstract Methods and Findings section, please describe the main limitation(s) of the study's methodology.
g)    Please remove “funding” statement

*These changes have been made*

6)    At this stage, we ask that you include a short, non-technical Author Summary of your research to make findings accessible to a wide audience that includes both scientists and non-scientists. The Author Summary should immediately follow the Abstract in your revised manuscript. This text is subject to editorial change and should be distinct from the scientific abstract. Please see our author guidelines for more information: <https://urldefense.proofpoint.com/v2/url?u=https-3A__journals.plos.org_plosmedicine_s_revising-2Dyour-2Dmanuscript-23loc-2Dauthor-2Dsummary&d=DwIGaQ&c=eRAMFD45gAfqt84VtBcfhfEazhEXT91ASHynm_9f1N0&r=ux1gxZrJUkrsePP3OAna0bSOOK-a1b0eEuGLtaV_sTk&m=hDKWgdZsooa5faWDjNqDi5gW-KYy1Pzgw2iiQzgpW4CP31fGphv2ACo3Qt-GYlwQ&s=CNAA-FKGSzDwibDmlGpCTrNy3AIEQiHYbSzxACHE7yA&e=>

*Included*

7)    Introduction:
a)    Please remove the description of the methods from the last paragraph, i.e., “we examine PrEP use cascade… refill data from the DREAMS database.” This can be moved to the Methods section.
b)    Please conclude the Introduction with a clear description of the study question or hypothesis.

*Done – the suggested text has been moved and the study questions are now stated at the of the introduction. The objectives addressed in the analysis are also now more clearly stated in the methods.*

8)    Did your study have a prospective protocol or analysis plan? Please state this (either way) early in the Methods section.

T*he study protocol did not include a detailed analysis plan. However, the analysis approach was consistent with one of our objectives, identifying predictors of persistence. Note that we expected to also analyze predictors of adherence but had too few adherent cases to pursue that line of inquiry.*

a)    If a prospective analysis plan (from your funding proposal, IRB or other ethics committee submission, study protocol, or other planning document written before analyzing the data) was used in designing the study, please include the relevant prospectively written document with your revised manuscript as a Supporting Information file to be published alongside your study and cite it in the Methods section. A legend for this file should be included at the end of your manuscript.
b)    If no such document exists, please make sure that the Methods section transparently describes when analyses were planned, and when/why any data-driven changes to analyses took place.
c)    In either case, changes in the analysis-- including those made in response to peer review comments-- should be identified as such in the Methods section of the paper, with rationale.

*A sentence has been added to the methods explaining why we did not examine predictors of adherence as originally planned.*

9)    Please ensure that the study is reported according to the STROBE and include the completed STROBE checklist as Supporting Information.

Please add the following statement, or similar, to the Methods: "This study is reported as per the Strengthening the Reporting of Observational Studies in Epidemiology (STROBE) guideline (S1 Checklist)."
a)    When completing the checklist, please use section and paragraph numbers, rather than page numbers.

*Included in the supplementary materials.*

10)    Statistical analysis
a)    Please include adjustment variables.

*The multivariable models adjusted for county of residence and factors that had a p-value<0.1 in the univariable analysis. Variables included in multivariable models are shown in Tables 2,S4 and S5.*

b)    Please adjust for clustering at individual and facility level.

*Adjustment has been included at the individual and ward level. We do not have facility level data; individual wards are small administrative units – a ward is much smaller than county but may contain several safe spaces (facilities). The univariable and multivariable generalized estimating equations with logit link in the models adjusted for clustering of study participants at the ward level. The outcome of interest (persistence) was not measured repeatedly for each participant and no further individual-level adjustment was made.*

11)    Please provide p values in addition to 95% CIs in the main text and tables

*Done*

12)    Please define all abbreviations in Tables and Figures e.g., PrEP, LTFU, TFV-DP, DBS, AGYW

*Abbreviations have been defined in all tables and figures.*

*PrEP = pre-exposure prophylaxis; LTFU=lost to follow-up; TFV-DP=tenofovir-diphosphate; DBS=dried blood spot; AGYW=adolescent girls and young women.*

13)    Please indicate in the figure caption the meaning of the bars and whiskers in Figure S1.

*Figure S1 has been replaced with a table (TableS4) which more clearly shows the data now.*

14)    Please replace the term “condom users” with “AGYW who use condoms”

*Done*

15)    Please remove the ‘Declaration of interest statement” and “Data sharing” from the end of the main text. In the event of publication, this information will be published as metadata based on your responses to the submission form.

*Done*

16)    References:
a)    Please select the PLOS Medicine reference style in your citation manager. In-text reference call outs should be presented as follows noting the absence of spaces within the square brackets, e.g., "... services [1,2]."
b)    References should have six names before et al. For those with more than six names, please ensure that et al., is inserted after six names
c)    Please ensure that journal name abbreviations consistently match those found in the National Center for Biotechnology Information (NCBI) databases. <https://urldefense.proofpoint.com/v2/url?u=https-3A__journals.plos.org_plosmedicine_s_submission-2Dguidelines-23loc-2Dreferences&d=DwIGaQ&c=eRAMFD45gAfqt84VtBcfhfEazhEXT91ASHynm_9f1N0&r=ux1gxZrJUkrsePP3OAna0bSOOK-a1b0eEuGLtaV_sTk&m=hDKWgdZsooa5faWDjNqDi5gW-KYy1Pzgw2iiQzgpW4CP31fGphv2ACo3Qt-GYlwQ&s=NHU9SdBnRRjosJXGrarVddOSrErMaEmHdzKOfkDHkd8&e=> .
d)    Ref #20 is incomplete

*Done, formatting of references has been changed as suggested. Prior reference 20 has been deleted.*


Comments from the reviewers:

Reviewer #1: The manuscript by Tabsoba, et al. report on the "real-world" use of oral PrEP among AGYW in two counties in western Kenya with a high incidence of HIV. Please see my detailed comments below about each section.

Introduction:
*    The statement on new HIV infections in the two countries comes from a 2015 reference, seven years ago. The authors should update this number with a more recent reference.

*References updated*

*    I would not consider the 3-month visit "long-term adherence"

*‘long-term’ has been removed*

*    How were the 336 participants selected from the larger group in the DREAMS PrEP program—either explain in introduction, or remove this detail and leave for the methods section

*Removed from the introduction; a more complete explanation of participant selection has been added to methods section*.

Methods:
*    This sentence doesn't make sense to me: "and a two-level factor potentially associated with PrEP adherence."

*Wording has been modified for clarity*

*    I realize that this study was conducted in a real-world setting, but anticipating a 50% loss to follow-up is very high

*We agree that this may be high, but it was the level used to calculate the sample size. The assumed 50% rate was for the combined rate for loss to follow up and rate for discontinuation. This assumption was based on initial DREAMS programmatic data in the early stage of the roll out in Kisumu and Homa Bay counties in Kenya.*

*    I'm confused about the methods of sampling. In one section it is mentioned that a random sample was obtained from the original enrolled cohort in the DREAMS PrEP program. However, the methods used to randomly sample are not provided.

*The method (Stata ‘sample’ command) used for selection of random sample has been added to method section.*

*Secondly, a flow chart would help the reader to assess what happened along the enrolment cascade, who was subsequently excluded from the analysis (e.g., they failed to return for follow-up, and/or didn't report taking PrEP) would be tremendously helpful.

*A flow chart has been added as figure 1 to clarify the enrollment and PrEP persistence cascade.*

*In the Results section (first paragraph) there is new mention about who was eligible—higher risk sexual behaviors. This information makes once think that random sampling was not deployed. I think that these are the criteria that were used to enroll the DREAMS cohort but can't be certain. Again, this section should be in the Methods not the Results section.

*The information in the first paragraph of the results refers to the eligibility criteria for the DREAMS PrEP program (not our study). This has been moved to the methods section.*

Results:
*    "Kisumu, the remainder in Homa Bay." Does this refer to the city/town or counties?

*This refers to counties and has been added.*

*    "PrEP program for a mean of 142 days." Provide SD—or is median a better measure with IQR?

*Median and IQR have been added. The median time in the PrEP program was 112 (IQR: 90-172] at enrollment.*

*    Throughout Results it would be helpful to the reader to provide the Ns along with the percentages.

*Done*

*    "PrEP persistence," not sure this is the standard designation in the literature

*We have not found a consistent standard definition of persistence in our review of the literature. Most definitions include attendance at visits without consideration of adherence to PrEP use; several examples are listed below including one which includes consideration of client’s desire to continue PrEP (included in our definition as well).*

1. *Persistence = Attending the program in the past 6 months (without consideration of adherence) [1]*
2. *Persistence = Maintaining refills in PrEP care (1^st^ year, 2^nd^ year, first 2 years) [2].*
3. *The complement of PrEP discontinuation (i.e., ‘survival’ from discontinuation) = PrEP persistence*

*where discontinuation was defined as a composite outcome based on a) not attending two consecutive visits (not returning for PrEP), b) attending a monthly appointment and the client choosing to discontinue PrEP (client discontinuation), or c) attending a monthly appointment and the clinician deciding to discontinue PrEP (provider discontinuation) [3].*

*We believe that these definitions are largely consistent with our definition. The definition in our paper (under results section) reads:*

*This left 176 of the 302 AGYW who attended Interview 2 (58.3%, 95%CI [52.7%, 63.8%]) who met* ***our definition of PrEP persistence,*** ***having attended both the second interview and an interim refill visit (Figure 1 and 2A, Table S1 panel A).***

*    Suggest adding "among" to "in contrast to 38/105 (36%) 'among' discontinuers."

*Done*

*    Table 1 has quite several errors—second column is not all %s, use of "****", etc. It also could benefit from reformatting

*Done*

*    Why was the US Household Security Survey used? The validated HFIAS has been used repeatedly in the same population in western Kenya

*We agree that the scale used is not appropriate and have removed these data from the paper*

*    I'm not sure that Figure 2 adds much more than what is stated in the narrative

*This figure has been moved to the supplemental material.*

Discussion:
*    The discussion section is well written in general, with adequate references to substantiate the relevance of the new data from the study. In addition, the authors do spell out some of the weaknesses, while acknowledging its strengths. My primary concern is with generalizability of these data, based on some of the weaknesses I identified with the methods, or at least the description of the methods used.

*We have clarified the methods used (especially those related to participant selection) and hope that these clarifications address the weakness cited and the concerns about generalizability.*


Reviewer #2: Statistical review

This paper reports an observational study that investigates factors associated with persistence in a PrEP programme amongst adolescent girls and young women. I had some comments on the statistical methods/reporting of the study:

1.    Abstract "samples taken at the second visit" - it might be useful if the same language as the previous sentence is used - is second visit the same as the second interview or the interim refill visit?

*The wording has been changed throughout to clarify that the second visit is the second interview.*

2.    Methods, analysis "Persistent AGYW were defined as having attended both interviews as well as interim visits for PrEP refills and stating that they were taking PrEP at both interviews. Those lost to follow up between visits were excluded from analysis." - I found this confusing whether those lost to follow-up were included as non-persistent or excluded, or is this saying that only persistent AGYW were included in analysis? If loss to follow-up led to exclusion, this seems to mean an important set of likely non-persisters was excluded?

*AGYW who were lost to follow-up (LTFU) were not combined with AGYW who attended the second interview but were excluded. These 18 AGYW represented only 5% of the original sample. While they are definitely not persistent, they may be quite different from AGYW who came to the second interview visit but said that they were not continuing PrEP (most of these AGYW also came to at least one refill visit). While we have Interview 1 data for AGYW who LTFU were, we do not have interview 2 data which was used in the analysis of persisters vs non-persisters.*

3.    Methods, analysis: can more be said about what characteristics were considered for the model - is it the ones in study procedures? Some of these wouldn't vary between visit 1 and visit 2 but others would - I wasn't sure why visit 2 characteristics were used instead of visit 1 characteristics? Using visit 1 characteristics would strike me as being more about predicting which participants are persistent.

*Variables with p values <.1 in univariable analyses were included in the multivariable analyses. While visit 1 characteristics would be more useful in predicting which participants will be persistent, the analysis we conducted focused on characteristics that were considered for their plausible associations with persistence at the contemporaneous timepoint. Importantly, the variable that was most strongly associated with persistence (perceived moderate to high risk of HIV if not taking PrEP) was measured at interview 2, but not at Interview 1. We have included below the results of an analysis looking at associations between persistence and factors assessed at the first interview.*

Discontinuers versus Persisters - Factors measured at Interview 1 associated with PrEP persistence

|  | **Discontinuers**  **(N=105)** | **Persisters**  **(N=176)** | **Univariable analysis^1^** | | **Multivariable analysis^2^** | |
| --- | --- | --- | --- | --- | --- | --- |
| **Factor** | **n** | **n** | **Odds ratio OR[95%CI]** | **p-value** | **Odds ratio OR[95%CI]** | **p-value** |
| Age ≥22 years | 56 | 119 | 1.78 [ 1.04 , 3.04 ] | 0.035 | 1.56 [ 0.82 , 2.98 ] | 0.175 |
| Currently in school | 45 | 55 | 0.59 [ 0.4 , 0.89 ] | 0.011 | 0.69 [ 0.4 , 1.22 ] | 0.205 |
| In PrEP support group | 81 | 142 | 1.28 [ 0.62 , 2.64 ] | 0.496 |  | . |
| **Currently has multiple sex partners** | 10 | 35 | 2.42 [ 1.57 , 3.72 ] | <0.001 | 3.27 [ 2.45 , 4.36 ] | <0.001 |
| Married/cohabiting | 44 | 82 | 1.18 [ 0.71 , 1.97 ] | 0.515 |  | . |
| One or more children | 68 | 132 | 1.62 [ 0.96 , 2.72 ] | 0.069 | 0.72 [ 0.39 , 1.33 ] | 0.293 |
| Lives with parents or grandparents | 54 | 79 | 0.78 [ 0.45 , 1.35 ] | 0.376 |  | . |
| Lives with partner | 43 | 81 | 1.21 [ 0.71 , 2.06 ] | 0.489 |  | . |
| Partner is aware of PrEP use | 60 | 107 | 1.15 [ 0.74 , 1.77 ] | 0.539 |  | . |
| **Partner is very supportive of PrEP use** | 33 | 79 | 1.76 [ 1.34 , 2.32 ] | 0.000 | 1.95 [ 1.39 , 2.74 ] | <0.001 |
| Partner is HIV positive | 3 | 10 | 2.04 [ 0.71 , 5.87 ] | 0.188 |  | . |
| Partner has other partners | 33 | 70 | 1.44 [ 0.81 , 2.58 ] | 0.217 |  | . |
| AGYW believes partner puts her at risk | 47 | 91 | 1.35 [ 0.81 , 2.24 ] | 0.252 |  | . |
| Current HIV chance, moderate/high | 14 | 18 | 0.74 [ 0.42 , 1.32 ] | 0.311 |  | . |
| Experience of intimate partner violence | 13 | 36 | 1.82 [ 0.94 , 3.55 ] | 0.078 | 1.43 [ 0.77 , 2.66 ] | 0.259 |
| Depression, moderate to severe | 19 | 44 | 1.51 [ 0.73 , 3.11 ] | 0.265 |  | . |
| Social support (most or all the time) | 15 | 18 | 0.7 [ 0.29 , 1.69 ] | 0.428 |  | . |
| Inconsistent or no condom use | 82 | 126 | 0.7 [ 0.45 , 1.09 ] | 0.116 |  | . |
| Contraceptive use, any | 86 | 146 | 1.1 [ 0.59 , 2.03 ] | 0.773 |  | . |
| oral | 5 | 7 | 0.84 [ 0.28 , 2.5 ] | 0.752 |  | . |
| injectable | 19 | 32 | 1.03 [ 0.62 , 1.73 ] | 0.905 |  | . |
| Implant | 23 | 60 | 1.83 [ 0.93 , 3.58 ] | 0.079 | 1.33 [ 0.67 , 2.64 ] | 0.408 |
| **male condoms** | 38 | 42 | 0.56 [ 0.37 , 0.84 ] | 0.005 | 0.63 [ 0.41 , 0.97 ] | 0.036 |
| female condoms | 1 | 2 | 1.28 [ 0.12 , 14.16 ] | 0.842 |  | . |
| Months since PrEP initiation at interview1, 2-3 months | 30 | 45 | 0.87 [ 0.43 , 1.75 ] | 0.693 |  | . |
| Months since PrEP initiation at Interview 1, 4-6 months | 59 | 96 | 0.92 [ 0.6 , 1.42 ] | 0.714 |  | . |
| Months since PrEP initiation at Interview1, 6 + months | 15 | 35 | 1.49 [ 0.74 , 3 ] | 0.262 |  | . |
| Education, primary school | 32 | 61 | 1.21 [ 0.72 , 2.04 ] | 0.477 |  | . |
| Education, secondary school | 57 | 96 | 1.02 [ 0.59 , 1.75 ] | 0.951 |  | . |
| Education, postsecondary | 16 | 19 | 0.66 [ 0.29 , 1.5 ] | 0.325 |  | . |

**^1^Odds ratio and corresponding p-value were based on univariable generalized estimating equations with logit link function in the model accounting for clustering of study participants within wards.**

**^2^Odds ratio and corresponding p-value were based on multivariable generalized estimating equations with logit link function in the model adjusted for county of residence, factors with p-value<0.1 in the univariable analysis as well as clustering of study participants within wards.**

4.    Methods, analysis: can more be added about the stepwise procedures used to build the model (including rules for adding/deleting variables), perhaps in supplementary material? Standard approaches such as forward or backward selection have been criticised so it would be good if more than 1 approach led to the same variables being included, to demonstrate robustness. It may be worth considering a sparse regression approach to supplement this analysis.

*Results from step-wise models have been replaced by new analyses in tables 2, S4 and S5, where variables with p values <.1 in the univariable analyses were included in the multivariable analyses. The changes were made to present the results of both univariable and multivariable analyses and to better account for clustering of study participants within wards using generalized estimating equations with a logit link function in the models following requests from the editors.*

5.    Methods, analysis: in the results, censored individuals are mentioned - presumably these were excluded from analysis?

*Yes. The fact that the 16 who were censored are excluded is now presented in the text and in Figure 1.*

6.    Table 2 and Figure 2 - I didn't follow why the p-values were calculated comparing discontinuers and persisters who were taking no PrEP. This doesn't seem consistent with the hypothesis that the study was powered for in the sample size calculation: factors related to PrEP adherence.

*Although the sample size calculations were based on adherence, the analyses presented address two study objectives relating to adherence and persistence. This is now stated in the methods ;” This analysis focuses on two study objectives: measurement of PrEP adherence by self-report and biomarkers, and identification of factors associated with PrEP persistence and adherence.” We present findings related to adherence by self-report and PrEP metabolite levels. Due to the very low levels to objective PrEP use and the discordance between these values and self-report, we have focused much of our analysis on persistence and factors associated with persistence.*

7.    I think Figure S1 would be better included in the main paper.

*Figure S1 has been deleted. The multivariable analysis is now included in Tables 2, S4 and S5.*

James Wason


Reviewer #3: Thank you for the opportunity to review this manuscript that describes an assessment of PrEP adherence in 336 AGYW participating in a DREAMS programme in Western Kenya who attended two study visits.

Major comments
1.    The manuscript would benefit from a figure that shows the study design and or the attrition of participants at each step of the analysis. Participants appear to be excluded at certain points but the rationale for non-inclusion was not entirely clear as those with some information may provide important insights.

*This figure is now provided as figure 1. We have also included text in the Methods section describing how the AGYW who were lost to follow-up or censored have been treated in the analysis.*

2.    The primary outcome appears to be PrEP adherence defined as TDF/FTC levels on DBS >700f/ml per punch. The authors appear to use a variety of cut-offs however and it is not always clear how these were chosen. In the methods it would be helpful to provide a brief summary of the cut offs and the reasons selected based on published evidence as well as how these correspond with dosing (and limitations given largely based on MSM data). DBS reflects use in the past 4 weeks, but cut-offs may vary depending on whether this is first 4 weeks of use or not. The authors may want to be more clear about these time frames and the relation between outcome assessments and timeframes for reporting exposures/factors associated with adherence.

*Tenofovir-diphosphate (TFV-DP) has an relatively long half-life of 17 days and thus accumulates appreciably in red blood cells with repeated daily doses [4]. Measurement of TFV-DP can thus be used to assess cumulative patterns of adherence over longer time periods (weeks to months). High concentrations of tenofovir-diphosphate (TFV-DP) measured in dried blood spots (>700 fmol/punch) also correlated with substantial reduction in HIV risks [5-7].*

*Adherent AGYW were defined as having TFV-DP levels of 700+ fmol/3mm punch, a level achieved by both men and women taking 4+ doses per week [7] which is associated with protection from HIV infection. We also stratified TFV-DP levels above and below 350 fmol/punch for analyses, which roughly correlates with taking TDF on two days per week [7]. Studies of the correlation of TFV-DP levels to daily dosing have assumed consistent dosing to achieve steady state, which is achieved within 8 weeks [7]. Note that by design, all AGYW were in the PrEP program for at least this long prior to the first DBS sample. Despite this, the extremely low levels we observed suggest that almost all AGYW were not at steady state dosing of 2 or more doses per week (350 fmol/punch). Thus, we have also included the lower limit of TFV-DP quantification (200 fmol/punch) and the lower limit of detection (10 fmol/punch).  We have used the level of detection (10 fmol/punch) in our primary analysis to distinguish AGYW taking PrEP at least occasionally from those with no PrEP use in the recent past.*

3.    The authors argue that they have defined a PrEP cascade; however the definitions are somewhat different to definitions published by other authors on the PrEP cascade. It would be helpful if the authors could better define the process of the DREAMS PrEP programme, e.g. how initiation occurs, how often refills are given, frequency of HIV testing etc. Then the authors can locate their study on top of this framework and provide their definition of persistence which appears to be related to study visits primarily, with the requirement to return for at least one PrEP refill. The authors could perhaps explain why visits were not aligned with PrEP refill visits for AGYW as that could assist with anchoring their recall/reporting better perhaps. The authors may wish to include a brief review of PrEP cascades in the introduction to underpin their choice of terminology or methods.

*See response to Reviewer 1 for discussion of our definition of persistence. A review of PrEP cascades is not included in the introduction due to space considerations.*

*Process of DREAM PrEP programme: Engagement with the PrEP program begins with ‘PrEP sensitization’, an information session led by either a PrEP provider or the DREAMS mentor. The mentor than administers (or if literate) the AGYW self-administers a PrEP eligibility assessment (this includes the 7 eligibility criteria listed in the paper.) If eligible, the AGYW is referred to the clinic where she undergoes HIV and liver/kidney function tests, further PrEP education and is enrolled on PrEP. She returns monthly for refill visits, which sometimes take place at the Safe Space, but may also require a return visit to the comprehensive care clinic. AGYW who continue with PrEP may be shifted to a schedule for refill visits every 3 months (though few of the AGYW in our study were on such a schedule). HIV testing is expected to happen every 3 months.*

*The study interviews largely aligned with visits by the AGYW to the DREAMS program, but additional outreach occurred if AGYW were not contacted at these interview visits.*

4.    The authors use several measures in the baseline assessment as well as for assessments at the second visit. These are not defined in the methods but only as footnotes to tables. It would be helpful to define these measures more clearly in the methods section and reasons for use/validation of these measures in adolescent populations in sub-Saharan Africa. For example, is it appropriate to use a US-based food security measure in the Kenyan context? When defining measures add scales, cut-offs and whether or not validated in this or similar young African populations.

*We agree that the use of food security scales developed for use in US populations is inappropriate in our Kenyan study population. We have deleted these data. We have added additional references citing use of the scales we used in similar populations in Africa. Due to word limits, we have left the descriptions of the measures used in the Table 1 footnotes. With the editors’ permission, these could be moved to the text.*

*The HITS (Hurt, Insult, Threaten, Scream) scale to measure intimate partner violence is a 4-item scale that has been validated in numerous settings and with different populations, and used in studies with AGYW in Kenya [8, 9].*

*The Medical Outcomes Study (MOS) Social Support Scale includes 19 items [10] and has also been employed in cross-cultural contexts, including in South Africa, Kenya, and Uganda [11-13].*

*The PHQ-9 contains 10 items and has been used and validated among HIV positive adults, though not adolescents, in Kenya [14]. A recent study found the PHQ 9 to be acceptable among pregnant and postpartum women in Kenya [15].*

*The AUDIT scale (10 items) is widely used and was originally developed by the World Health Organization using multi-country data that included Kenyan participants [16].*

5.    The authors do not describe the package of interventions offered to AGYW to support their PrEP adherence apart from a brief mention in table 1 regarding a PrEP support group. There is value in understanding what if any adherence support interventions were offered and how they aligned with PrEP refill visits to provide context to the environment in which PrEP was he.

*Efforts within DREAMS to support adherence among AGYW include reminder messages from mentors to return for refill visits, and membership in PrEP support groups, for AGYW who elected to participate. Providers may also provide adherence counseling at refill visits.*

6.    The authors used an interviewer administered tool to solicit information from participants. Could they comment further of strategies to minimise social desirability bias. The participants seem to understand that they were being interviewed about their PrEP use. The authors observe the difference between reported and actual actions, but do not comment on the potential bias induced by the data collection process.

*Our results suggest strong social desirability bias influenced subjective measures of PrEP adherence. Training for interviewers emphasized the importance of establishing rapport and encouraging AGYW to speak freely. Questions were prefaced by statements encourage AGYW to be honest. Though the consent form included language to reassure participants that their answers would not be shared with providers or with the DREAMS program, we believe AGYW were reluctant to admit nonadherence. The possibility of social desirability bias is now mentioned in the discussion.*

7.    In the discussion, the authors appear to discount reasons for non-PrEP use as not related to PrEP. However, in reviewing table S2 it appears that many of the reasons for discontinuation are supply side or programmatic issues e.g. stock outs, lack of access because travelled from PrEP refill site or returned to school. In addition, side effects may also be an issue with poor counselling for those who are in early PrEP start up. The aggregation of PrEP users at different stages of PrEP use (less/more experienced) makes it challenging to tease apart some of these issues.

*Difficulties arising from stock-outs, travel etc. may be related to the PrEP program (which could be changed) but not directly related to PrEP itself (difficult or impossible to change). The wording in the discussion section has been modified for clarity.*

*All AGYW had to have been in the PrEP program at least 2 months at interview 1. They may not have been taking PrEP, but none were completely new to the program.*

8.    There are very few observations in the manuscript around the social and structural barriers to PrEP access for these young participants e.g. 17% experience IPV, 12% have adequate social support, 75% have food insecurity, most are married and have children and live in multi-generational homes. It is true that the data confirms that participants are motivated to use PrEP but are not able to act on those intentions perhaps because of the more pressing concerns in their life. It would be helpful if the authors could reflect on how these upstream factors may influence the direct factors observed to be associated with lack of persistence.

*AGYW’s intention to use PrEP as indicated by their “persistence” in the PrEP program, and by their self-reported intention to use,  did not correlate with actual use (as measured by protective levels).  While there may be several factors operating that contribute to this, our data did not show correlations/association with any specific upstream factors such as living in a multigenerational household (lack of privacy), social support, IPV, or depression. We are currently analyzing in-depth interview data collected concurrently, which may shed light on factors associated with lack of persistence and with poor adherence among persistent AGYW.*

9.    At times the sections bled into one another, methods in the results section, results in the discussion and discussion in results. The manuscript would benefit from a thorough edit to define each section more clearly and to make it easier for the reader to follow the arguments being made with the data.

*The manuscript has been edited to align text more closely with section headers*.

10.    In table 2, it is not clear what the P-value refers to (although the heading defines persisters vs continuers). Please clarify which comparison the p-value refers to with respect to the persister column, since those with detectable vs non-detectable PrEP appear quite different. It may be more helpful to present an analysis of continuers vs. discontinuers to understand barriers to PrEP continuation, and then to explore reasons for non-adherence which may not be the same as those for continuation and may require other interventions to address. Long-acting agents may help with the adherence issue, but may still be perplexed with the discontinuation challenge.

*The tables have been modified – Table 2 now presents univariate and multivariate analysis for persisters without evidence of PrEP use in the recent past to discontinuers. Table S4 presents a comparison of all persisters to discontinuers. P values in this table are presented more clearly.*

*We also include an analysis of non-persisters, including those who reported not taking PrEP and those without evidence of PrEP refills (105 + 21 =126) vs all persisters (176) as table S5. We believe this was what was requested as an analysis comparing continuers (i.e., persisters) to discontinuers (i.e., non-persisters). We have not included an analysis of reasons for non-adherence since almost all AGYW were non-adherent at Interview 2.*

11.    T

*We are not sure what this comment was meant to be*

Minor comments
12.    Abstract: define the outcome measures and cut offs for DBS as well as the assay in the methods, and the definitions used for levels consistent with no dosing/levels associated with protection.

*The abstract now states our definition of persistence as well as the levels consistent with protection and no dosing.*

13.    Abstract: define the persistence outcome in the methods of the abstract

*This is included: “176 (*58.3%*, 95%CI [*52.3-63.8*]) met our definition of PrEP persistence, having expressed intention to use PrEP, and attended both the second interview and an interim refill visit.”*

14.    Abstract: the conclusion states that this analysis identifies AGYW who may benefit from LA PrEP but it is not entirely clear from the findings how this population are identified without the use of a drug level assessment. Please clarify

*We have identified these AGYW using drug level assessment, but do not mean to suggest that this would be necessary in the future*. *With appropriate oral PrEP education, AGYW taking PrEP know whether they are adherent. Rather than widespread adherence screening to identify those not adherent, we propose that AGYW be given the option of LA PrEP if they find it difficult to comply with the oral PrEP regimen.*

15.    Introduction: TDF/FTC = tenofovir disoproxil fumarate/emtricitabine. Please spell out in full

*Done*

16.    Introduction: the authors should mention the PK requirement for near perfect PrEP dosing in women which may compound challenges with adherence and are different for MSM at least.

*We have not included discussion of PrEP PK due to limitations of space and scope and because the topic is rather complex. Studies have noted higher tissue concentrations of TFV and TFV-DP in rectal tissues compared to vaginal tissue after a single dose. In contrast, FTC concentrations were higher in the vaginal and cervical tissues than in the rectum, but FTC-TP concentrations declined more quickly [17]. A PK study in men and women taking directly observed therapy measured steady state blood levels of TFV-DP and FTC-TP and did not find appreciable differences by sex [7]. Finally, a review of PrEP studies in men or women found slightly lower effectiveness in women but concluded that differences were largely explained by lower adherence among women [18].*

17.    Introduction: please provide references for obstacles to adherence support in para 1 e.g. lack of partner support.

*Citations added*

18.    Introduction: CAB LA was evaluated in cisgender men and transgender women who has sex with men in the Americas, Thailand and South Africa and both HPTN 083 and 084 showed that CAB LA was superior to TDF/FTC for HIV prevention.

*Noted, included now in introduction*

19.    Introduction: clarify that plasma TDF/FTC was detectable in a random subset 56% of samples in HPTN 084 in women who had a median follow up of 1.2 years. The time frame seems relevant. In addition, injection coverage was across both arms of the study not just the CAB LA group. The sentence about visit attendance vs adherence probably needs to conclude this paragraph

*We have included the time frame for this study but now include the DBS data on TFV-DP (rather than plasma TDF/FTC which has a very short half-life) as it is a more appropriate comparator for our study. We have removed reference to women receiving CAB-LA in the sentence about injection coverage, since coverage was identical in both arms. An additional sentence about attendance and adherence has not been added due to word limitation..*

20.    Introduction: define persistence in relation to literature

*Clarification and references added. See also response to reviewer 1 and question 3 above for the definitions we and others have used.*

21.    Introduction: consider introducing a summary of the notion of a PrEP cascade and mention of the concept of Prevention effective adherence

*Due to word limitations, this has not been added.*

22.    Introduction: final comment appears to bleed into methods and may be better as a framing of objectives with definitions in methods

*This section has been removed and recapped in the method section.*

23.    Methods: would benefit from a figure that summarises time on PrEP and relation to sample collection and two visits/could summarise the reasons for inclusion/exclusion from analysis at each step of design

*This figure has been added as figure 1.*

24.    Methods: when defining measures add scales, cut-offs and whether or not validated in this or similar young African populations

*See response to reviewer #2. The Food security scale has been removed. In all cases, scales had not been validated with Kenyan AGYW specifically, though scales had been used in Kenya among other populations. Text in methods has been clarified.*

25.    Methods: what was the interval between study visits?

*The mean interval between the two interviews was 97 (SD=9) days and the median time between the two interviews was 93 (IQR [91,99]) days.*

*The second interview occurred a minimum of 3 months after the first. In a few cases, the interval was longer than 3 months as AGYW had returned to school.*

26.    Results: para 1; a description of the DREAMS PrEP programme including eligibility criteria for PrEP may be better in the methods since the reasons for non-inclusion are not related to these criteria it seems.

*This paragraph has been moved to methods section for clarity.*

27.    Results: can you provide some distribution of where respondents were in their PrEP journey with respect to the mean 142 days of PrEP? What % were in first month, months 2-3, months 3-6 as it might relate to a PrEP programme. This makes it easier then also to overlay where discontinuation might be happening.

*The median and IQR are now included in the text and Table 1*.

28.    Results: what was the mean interval between study visits?

*The median time between interviews among AGYW who attended Interview2 was 93 (IQR [91,99]) days.*

29.    Results: para 2 provide numerator, denominator and percentages so that it is possible to follow the logic of which group is being discussed.

*Done*

30.    Results: clarify lower limit of quantification stated as >199 f/mol per punch - this seems to contradict other statements and measures and may not be correct. Should this be defined in the methods?

*This is correct (199 fmol/punch is the lower limit of quantification) and has now been defined in the methods section.*

31.    Results: para that begins " Thus it seems…" seems to be editorial commentary and may be better as a comment in the discussion with a focus on presenting the outcomes in the results.

*This paragraph has been modified, including removal of the first two sentences.*

32.    Discussion: it’s not clear what data support the statement that programme retention was good.
*“good” has been changed to moderate.*

33.    Discussion: the clinical trial referred to in para 2 was not a trial of PrEP but an assessment of uptake and acceptability and included an evaluation of adherence strategies. Perhaps the difference is not real world vs. not real word but the focus on adherence support for an adolescent population.
*Wording has been clarified as suggested.*

34.    Discussion: para 3, not clear what data support the statement that this shows a cascade of care in the sense of how cascades have been described in the literature. May need to refine or clarify.

*Sentence has been clarified, removing mention of a PrEP cascade.*

35.    Discussion: the authors mention that they had access to pharmacy refill data. It would be helpful to summarise these for the cohort and present in relation to when they were interviewed. The refill data are mentioned but data are not presented systematically.

*Data on refill visits are included below.*

Number of PrEP refill visits between the dates of the two interviews (N=302)

| PrEP refill visit # | **Discontinuers** | **Continuers** |
| --- | --- | --- |
| 0 | 35 (33%) | 21 (11%) |
| 1 | 17 (16%) | 26 (13%) |
| 2 | 23 (22%) | 32 (16%) |
| 3 | 27 (26%) | 102 (52%) |
| 4 | 3 (3%) | 16 (8%) |

Number of days between the two interviews, Median [IQR]: 93 [91,99]

36. References: update to reflect published manuscripts where possible; some conference abstracts are referred to when in fact peer-reviewed manuscripts are available.
*Done*

**References for Response to Reviewers Document**

1.	Rolle C-P, Onwubiko U, Jo J, Sheth AN, Kelley CF, Holland DP. PrEP implementation and persistence in a county health department setting in Atlanta, GA. AIDS and Behavior. 2019;23(3):296-303.

2.	Coy KC, Hazen RJ, Kirkham HS, Delpino A, Siegler AJ. Persistence on HIV preexposure prophylaxis medication over a 2‐year period among a national sample of 7148 PrEP users, United States, 2015 to 2017. Journal of the International AIDS Society. 2019;22(2):e25252.

3.	Rao A, Mhlophe H, Comins C, Young K, Mcingana M, Lesko C, et al. Persistence on oral pre-exposure prophylaxis (PrEP) among female sex workers in eThekwini, South Africa, 2016–2020. PLoS ONE. 2022;17(3):e0265434.

4.	Anderson PL, Glidden DV, Liu A, Buchbinder S, Lama JR, Guanira JV, et al. Emtricitabine-tenofovir concentrations and pre-exposure prophylaxis efficacy in men who have sex with men. Science Translational Medicine. 2012;4(151):151ra25-ra25.

5.	Castillo-Mancilla JR, Zheng J-H, Rower JE, Meditz A, Gardner EM, Predhomme J, et al. Tenofovir, emtricitabine, and tenofovir diphosphate in dried blood spots for determining recent and cumulative drug exposure. AIDS research and human retroviruses. 2013;29(2):384-90.

6.	Grant RM, Anderson PL, McMahan V, Liu A, Amico KR, Mehrotra M, et al. Uptake of pre-exposure prophylaxis, sexual practices, and HIV incidence in men and transgender women who have sex with men: a cohort study. The Lancet infectious diseases. 2014;14(9):820-9.

7.	Anderson PL, Liu AY, Castillo-Mancilla JR, Gardner EM, Seifert SM, McHugh C, et al. Intracellular tenofovir-diphosphate and emtricitabine-triphosphate in dried blood spots following directly observed therapy. Antimicrobial agents and chemotherapy. 2018;62(1):e01710-17.

8.	Sherin KM, Sinacore JM, Li X-Q, Zitter RE, Shakil A. HITS: a short domestic violence screening tool for use in a family practice setting. Family Medicine - Kansas City. 1998;30:508-12.

9.	Sila J, Larsen AM, Kinuthia J, Owiti G, Abuna F, Kohler PK, et al. High awareness, yet low uptake, of pre-exposure prophylaxis among adolescent girls and young women within family planning clinics in Kenya. AIDS Patient Care and STDs. 2020;34(8):336-43.

10.	Sherbourne CD, Stewart AL. The MOS social support survey. Social Science and Medicine. 1991;32(6):705-14.

11.	Ncama BP, McInerney PA, Bhengu BR, Corless IB, Wantland DJ, Nicholas PK, et al. Social support and medication adherence in HIV disease in KwaZulu-Natal, South Africa. International Journal of Nursing Studies. 2008;45(12):1757-63.

12.	Nagata JM, Fiorella KJ, Salmen CR, Hickey MD, Mattah B, Magerenge R, et al. Around the table: Food insecurity, socioeconomic status, and instrumental social support among women living in a rural Kenyan island community. Ecology of Food and Nutrition. 2015;54(4):358-69.

13.	Tsai AC, Bangsberg DR, Emenyonu N, Senkungu JK, Martin JN, Weiser SD. The social context of food insecurity among persons living with HIV/AIDS in rural Uganda. Social Science and Medicine. 2011;73(12):1717-24.

14.	Monahan PO, Shacham E, Reece M, Kroenke K, Ong’Or WO, Omollo O, et al. Validity/reliability of PHQ-9 and PHQ-2 depression scales among adults living with HIV/AIDS in western Kenya. Journal of General Internal Medicine. 2009;24(2):189-97.

15.	Velloza J, Njoroge J, Ngure K, Thuo N, Kiptinness C, Momanyi R, et al. Cognitive testing of the PHQ-9 for depression screening among pregnant and postpartum women in Kenya. BMC Psychiatry. 2020;20(1):1-14.

16.	Babor TF, Higgins-Biddle JC, Saunders JB, Monteiro MG. The alcohol use disorders identification test (AUDIT): World Health Organization; 2001. Available from: <http://apps.who.int/iris/bitstream/handle/10665/67205/WHO_MSD_MSB_01.6a.pdf;jsessionid=7E01DD845099522D8F58D7A4560BA1F1?sequence=1>.

17.	Patterson KB, Prince HA, Kraft E, Jenkins AJ, Shaheen NJ, Rooney JF, et al. Penetration of tenofovir and emtricitabine in mucosal tissues: implications for prevention of HIV-1 transmission. Science Translational Medicine. 2011;3(112):112re4-re4.

18.	Fonner VA, Dalglish SL, Kennedy CE, Baggaley R, O’reilly KR, Koechlin FM, et al. Effectiveness and safety of oral HIV preexposure prophylaxis for all populations. AIDS. 2016;30(12):1973.
